# Supplementary material for: Organ-specific genome diversity of replication-competent SARS-CoV-2
Source: Nat Commun. 2021 Nov 16;12:6612. doi: 10.1038/s41467-021-26884-7 (PMC8595628; doi:10.1038/s41467-021-26884-7)
Supplement: Supplementary file 1 — Supplementary Information [file 41467_2021_26884_MOESM1_ESM.pdf]

Supplementary Information

Organ-specific genome diversity of replication-competent SARS-CoV-2

Jolien Van Cleemput, Willem van Snippenberg, Laurens Lambrechts, Amélie Dendooven, Valentino D'Onofrio, Liesbeth Couck, Wim Trypsteen, Jan Vanrusselt, Sebastiaan Theuns, Nick Vereecke, Thierry P.P. van den Bosch, Martin Lammens, Ann Driessen, Ruth Achten, Ken R. Bracke, Wim Van den Broeck, Jan Von der Thüsen, Hans Nauwynck, Jo Van Dorpe, Sarah Gerlo, Piet Maes, Janneke Cox, Linos Vandekerckhove

Supplementary Table 1

Case ID	Duration of disease in days	CCI	CCI subcategories											Invasive O_2	Immuno-suppressive therapy	Antiviral treatment	Viral RNA in lungs	RNAemia	Viral RNA in extrap organs
			CVD	CA	Str	HP	Dem	Dep	DPD	CKD	Diab	Dysl /Ob	Lymph						
01	21 (L)	3	1	0	0	0	0	0	1	0	0	1	0	Y	N	N	Y	Y	N
02	51 (L)	4	1	1	1	1	0	0	0	0	0	0	0	Y	N	N	N	N	N
03	41 (L)	4	1	0	0	0	1	0	1	1	0	0	0	N	N	N	N	Y	N
04	8 (S)	2	1	0	0	0	0	0	0	1	0	0	0	N	Y, mild[1]	N	Y	Y	N
05	27 (L)	2	1	0	0	0	0	0	0	1	0	0	0	N	N	N	Y	Y	N
06	18 (S)	3	1	0	0	0	0	0	1	0	0	0	1	N	Y, severe[2]	N	Y	Y	Y
07	3 (S)	1	0	0	0	0	0	0	1	0	0	0	0	N	Y, severe[3]	N	Y	Y	Y
08	18 (S)	4	1	1	0	0	0	1	0	1	0	0	0	N	N	N	Y	N	N
09	32 (L)	4	1	1	0	0	0	0	1	1	0	0	0	N	N	N	N	Y	N
10	28 (L)	4	1	1	0	0	0	0	0	1	0	1	0	N	N	N	N	N	N
11	>22 (L)	6	1	0	1	1	0	0	0	1	1	1	0	N	N	N	Y	N	N
12	12 (S)	1	1	0	0	0	0	0	0	0	0	0	0	N	N	N	Y	Y	N
13	88 (L)	1	0	0	0	0	0	0	0	0	0	0	1	Y	Y, severe[4]	Y, remdesivir[5]	Y	Y	Y
Summary	22 [18-32]	3 [2-4]	11/13	4/13	2/13	5/13	1/13	1/13	5/13	7/13	1/13	3/13	2/13	3 Y 10 N	3 Y 10 N	1 Y 12 N	9 Y 4 N	9 Y 4 N	3 Y 10 N

Table S1. Patient demographics and clinical data. Data are summarized in the bottom row as either median and [interquartile range] (age, duration of disease, CCI), absolute number (sex, invasive O_2, immunosuppressive therapy, antiviral treatment), or relative number (CCI subcategories). Abbreviations: M= male, F= female, S= short-lived disease, L= long-lived disease, Y= yes, N= no. CCI= Charlson Comorbidity index, CVD= cardiovascular disease, CA= cardiac arrythmia, Str= stroke, HP= hemiplegia, Dem= dementia, Dep= depression, CPD= chronic pulmonary disease, CKD= chronic kidney disease, Diab= diabetes, Dysl= dyslypidemia, ob= obesitas, Lymph= lymphoma. Footnotes: [1]=Sulfasalzine 500 mg long-term, [2]=Rituximab 500 mg/m^2 and methotrexate 3.5 gr/m^2 long-term, [3]= Methylprednisolone 8 mg long-term and 32 mg short-term, [4]=Ritixumab 500 mg/m^2 long-term and methylprednisolone 32-64 mg short-term. [5]=Three-day treatment with remdesivir 13 days prior to death.

Supplementary Figure 1

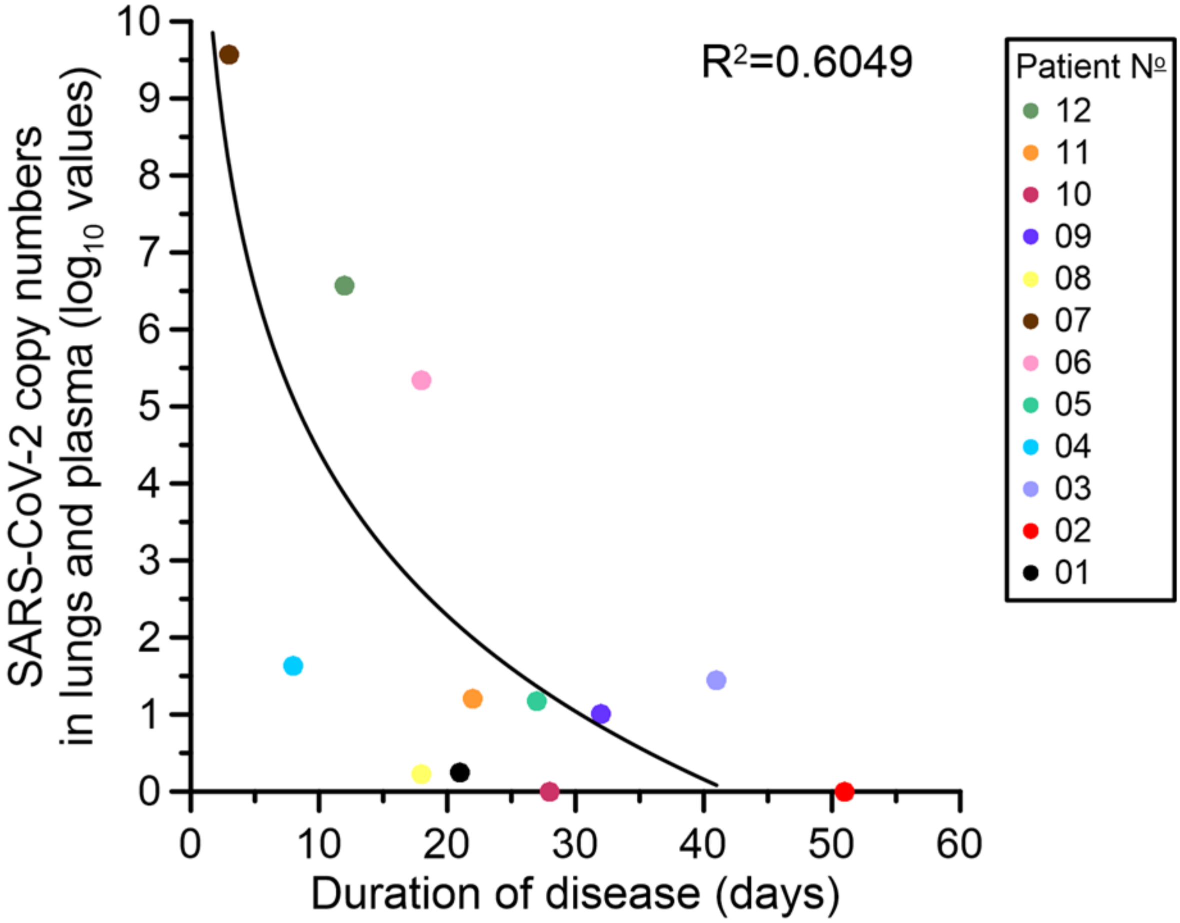

Figure S1. SARS-CoV-2 RNA loads in lungs and plasma are negatively correlated with duration of COVID-19 in fatal cases. The sum total of viral titers (log_{10} values) in lungs and plasma was plotted against duration of disease for each case (different colors). The solid line represents the logarithmic regression line (R^2= 0.6049, P= 0.003, F=15.308, df=10). Case 13 was considered an outlier and removed from the dataset. Source data are provided as a Source Data file.

Supplementary Figure 2

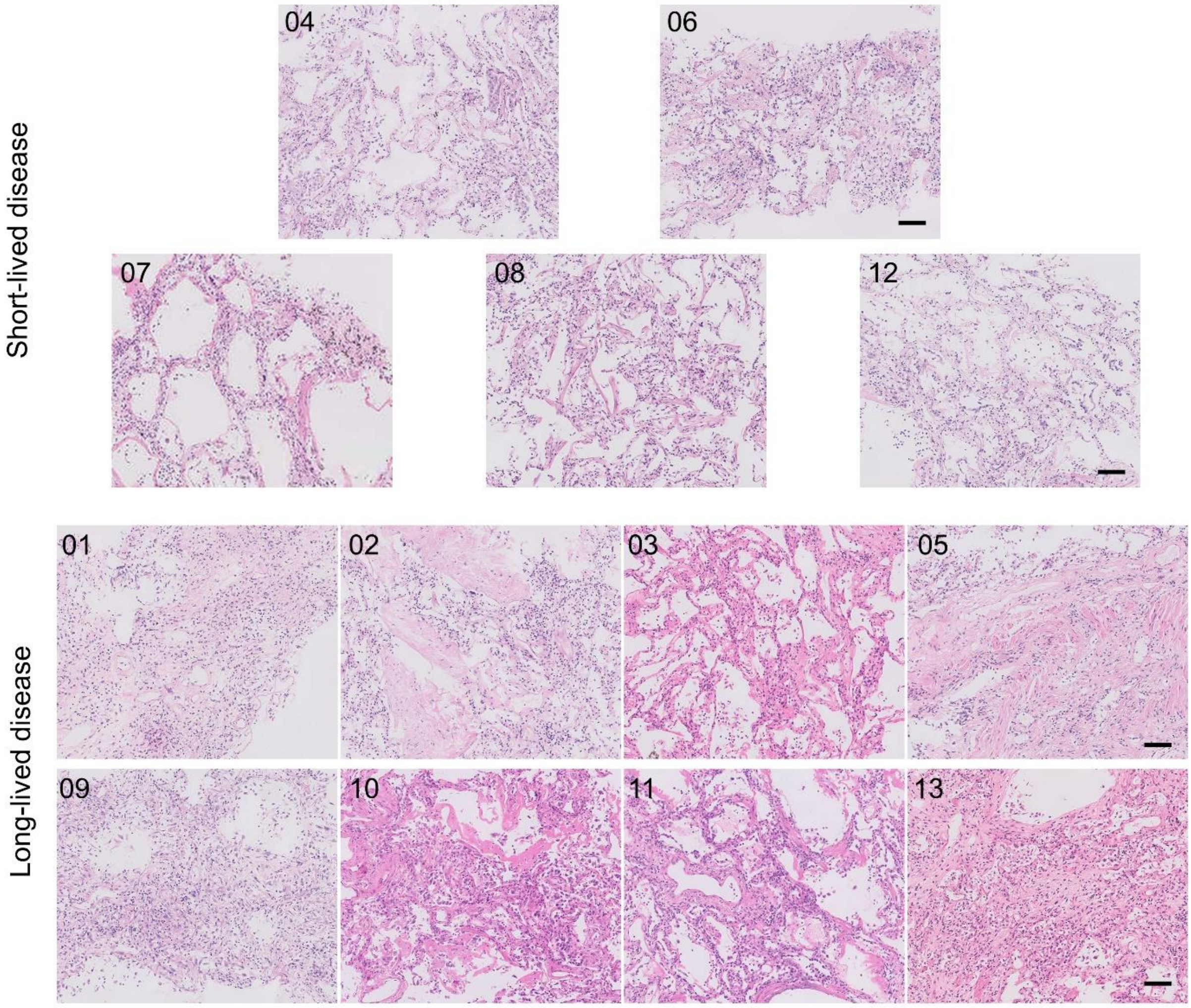

Figure S2. Histopathological patterns of lung biopsies. Hematoxylin (HE) staining of paraffin-embedded sections of lung tissues of our COVID-19 cohort. Cases with short-lived disease (№ 04, 06, 07, 08, and 12; upper panels) generally show histological patterns of acute exudative alveolar damage with reactive bronchial epithelia and edema, while cases with long-lived disease (№ 01, 02, 03, 05, 09, 10, 11 and 13; lower panels) are characterized by a remodeling pattern of acute respiratory distress with interstitial fibrosis and consolidation of airspace. Scale bars represent 100 µm.

Supplementary Figure 3

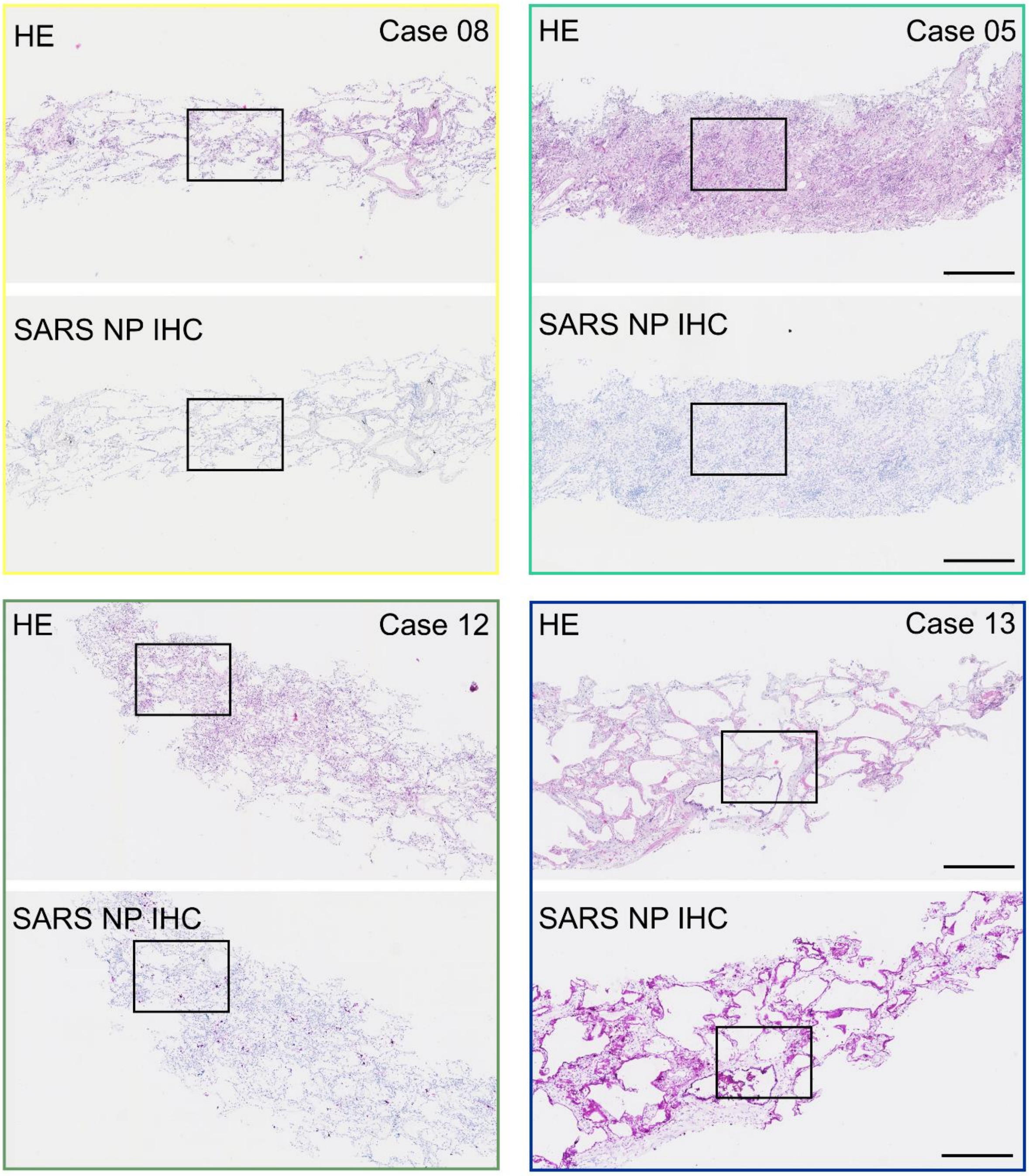

Figure S3. Lower magnification images of HE- and SARS-CoV-2 NP IHC-stained paraffin-embedded sections shown in Fig. 1b. Consecutive sections were either stained with hematoxylin-eosin (HE; upper images) or SARS-CoV-2 nucleocapsid protein (NP) immunohistochemistry (IHC; in purple; lower images). Picture delineations correspond to the respective case colors shown in the legend of Fig. 1a. The black squares represent selected areas shown in Fig. 1b (IHC) or corresponding areas on HE staining. Scale bars represent 500 µm.

Supplementary Figure 4

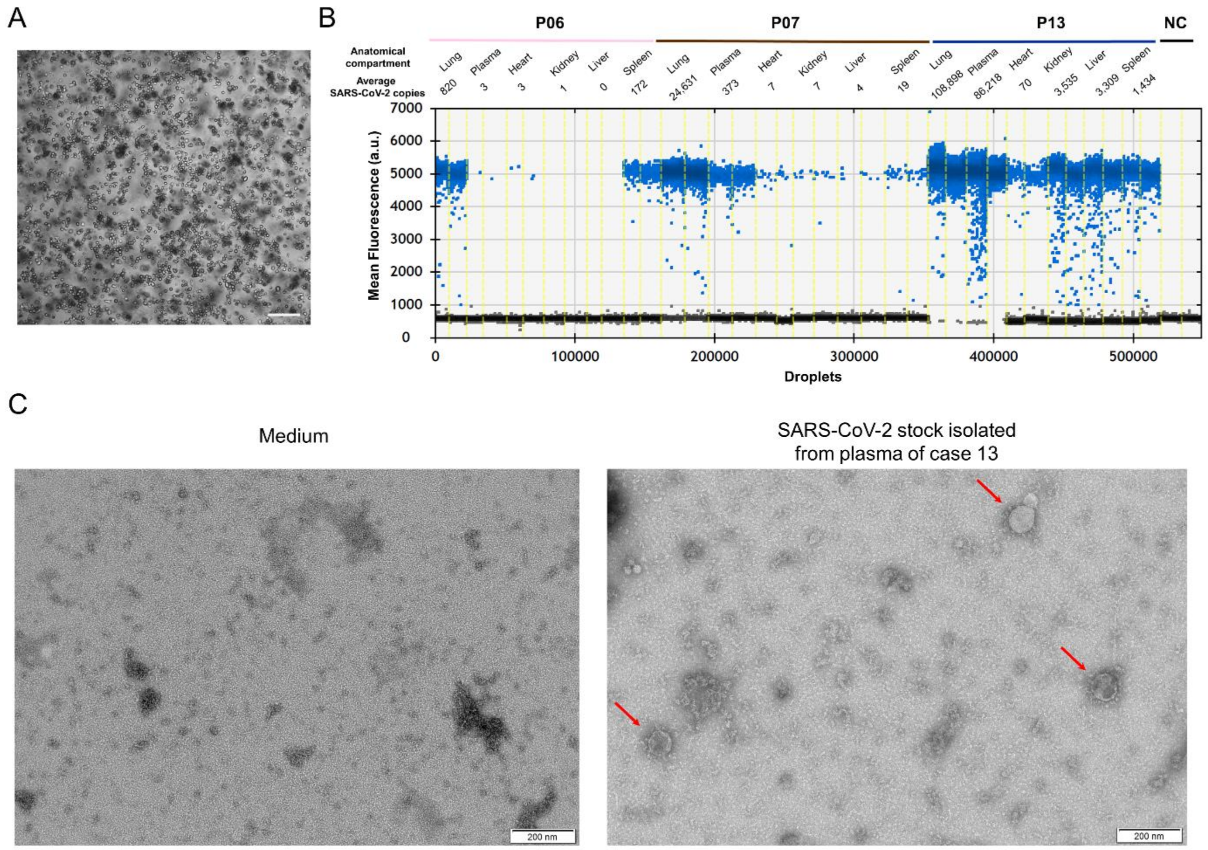

Figure S4. Systemic spread of SARS-CoV-2. (A) Inoculation of Vero E6 cells with SARS-CoV-2 from plasma of case 13 produced a lytic infection with characteristic rounding cytopathic effects (CPE) 2 days post inoculation. The scale bar represents 100 µm. (B) Tabulated absolute concentrations of SARS-CoV-2 genome copies in all organs and plasma of three patients with viral dissemination to multiple organs (case 06, 07 and 13) using ddPCR. (C) Transmission electron microscopy images of negative control (complete medium only) and plasma-derived viral progeny produced on Vero E6 cells. Arrows indicate virions and scale bars indicate 200 nm.

Supplementary Figure 5

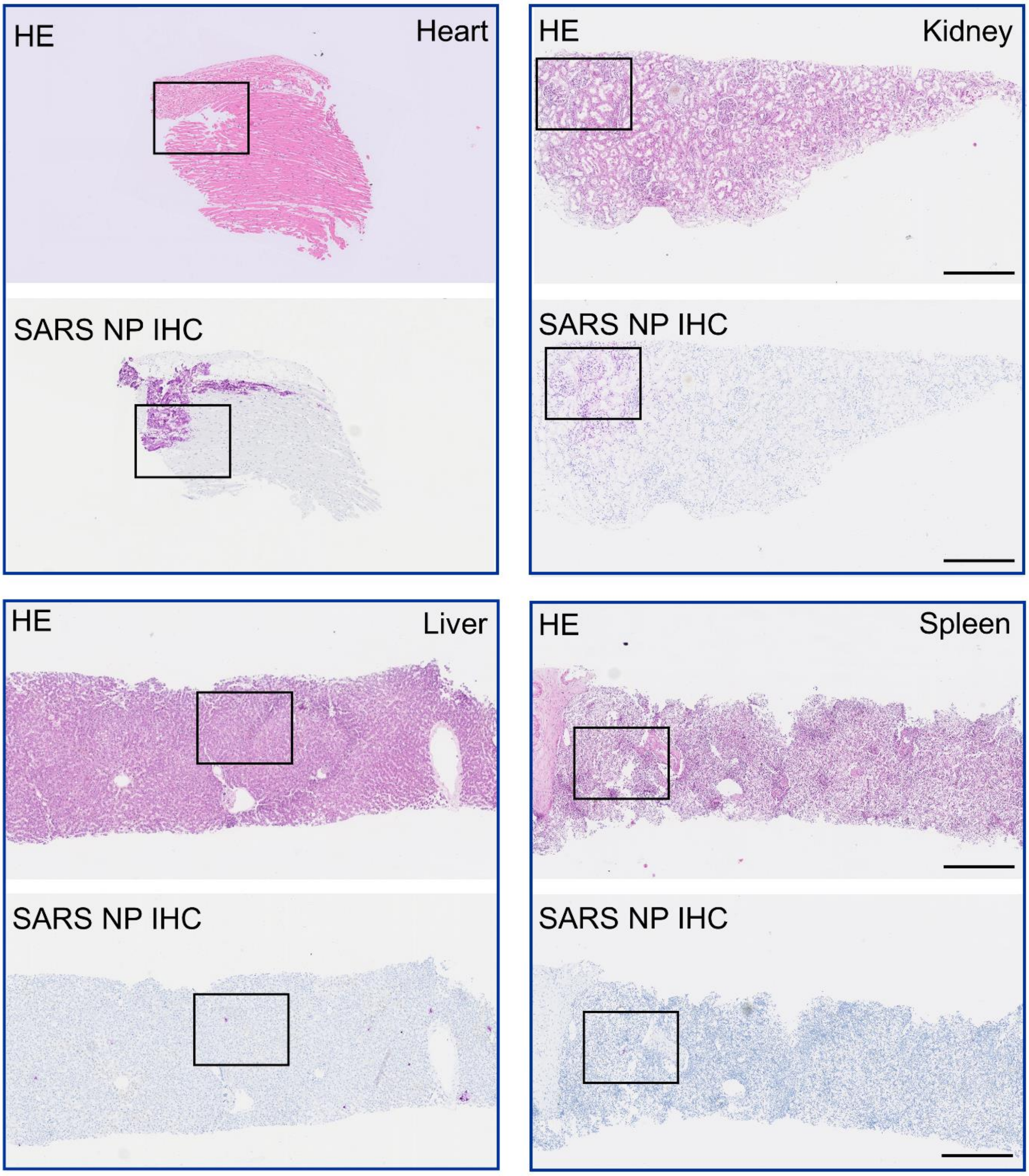

Figure S5. Lower magnification images of HE- and SARS-CoV-2 NP IHC-stained paraffin-embedded sections shown in Fig. 1e. Consecutive sections* of different extrapulmonary tissues were either stained with hematoxylin-eosin (HE; upper images) or SARS-CoV-2 nucleocapsid protein (NP) immunohistochemistry (IHC; in purple; lower images). Picture delineations correspond to the respective case colors shown in the legend of Fig. 1d. The black squares represent selected areas shown in Fig. 1e (IHC) or corresponding areas on HE staining. Scale bars represent 500 µm. *Cardiac tissue sections derived from the same tissue block, but were not consecutive.

Supplementary Figure 6

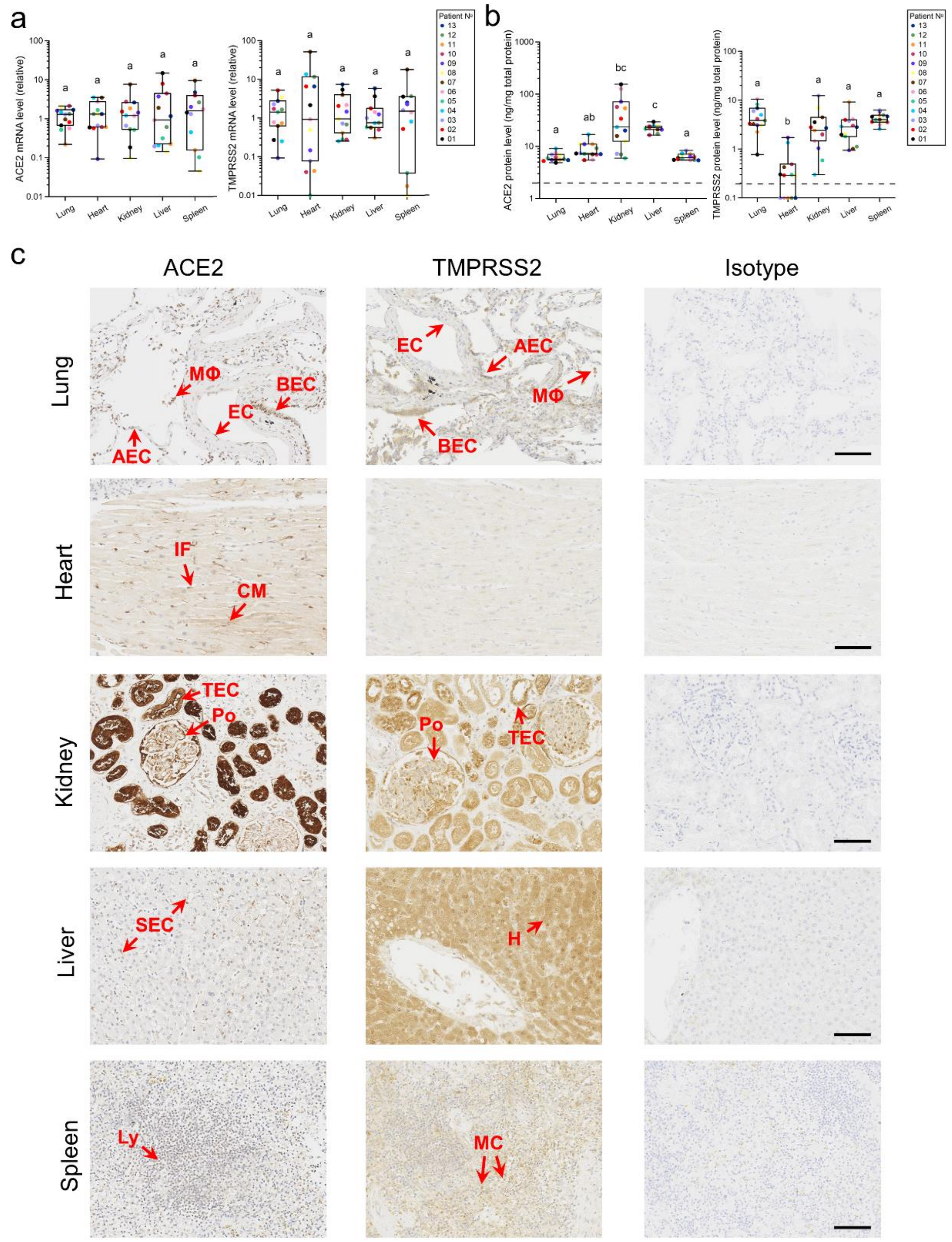

Figure S6. Mapping of ACE2 and TMPRRS2 expression in selected postmortem tissues of severe COVID-19 cases. Relative ACE2 and TMPRSS2 (a) mRNA and (b) protein levels in organs of different cases (n= 13, different colors). Central tendencies for mRNA and protein levels are illustrated as boxplots where the band indicates the median, the box indicates the first and third quartiles and the whiskers indicate the minimum and maximum of all of the data. One-way ANOVA was performed on log-transformed ACE2 mRNA levels and did not identify a significant difference in mean ACE2 mRNA values between different organs (P=1.000; F=0.000; df=63). A two-sided Kruskal-Wallis test did not identify a significant difference between TMPRSS2 mRNA values of different organs (P=0.999: df=4), but did identify significant differences between ACE2 and TMPRSS2 protein levels of different organs

(both P<0.001; df=4). Case 10 mRNA data were considered outliers and not included in the statistical test (n=12). The significance of pairwise two-sided comparisons (Bonferroni-corrected for the number of pairwise comparisons performed) is indicated by different letters on the plots. The P value for significant differences in ACE2 protein levels between different organs was <0.001, except for the comparison between heart and liver (P=0.034). The P value for significant differences in TMPRSS2 protein levels between different organs was <0.001 when comparing heart with lung and spleen and 0.014 and 0.012 when comparing heart with kidney and liver, respectively. Source data are provided as a Source Data file. (c) Representative images of ACE2 (long form only; left panels), TMPRSS2 (middle panels), and isotype control (right panels) staining (in brown) on paraffin-embedded sections of lung, heart, kidney, liver, and spleen tissue. Arrows indicate immune-stained cells. Abbreviations (in alphabetical order): AEC= alveolar epithelial cell, BEC= bronchiolar epithelial cell, CM= cardiomyocyte, EC= endothelial cell, H= hepatocyte, Ly= lymphocyte, Mϕ= macrophage, MC= myeloid cell, Po= podocyte, SEC= sinusoidal endothelial cell, TEC= tubular epithelial cell. Scale bars represent 100 µm.

Supplementary Table 2

Gene	Nt change	AA change	Frequency of detection per anatomical compartment (%)			
			Lungs	Plasma	Heart	Kidney
5' UTR	C241T	/	**99,86**	99,97	**99,97**	**99,97**
ORF1a	C398T	ORF1a:H45Y	**99,86**	99,75	**99,91**	**99,91**
ORF1a	C3037T	/	**99,88**	99,76	**99,72**	**99,84**
ORF1b	C13536T	/	**99,92**	99,92	**99,88**	**99,97**
ORF1b	C14408T	ORF1b:P314L	**99,6**	99,77	**99,7**	**99,56**
ORF1b	A20127G	/	**90,91**	100	**99,67**	**100**
S	C23731T	/	**100**	99,82	**99,28**	**98,96**
**Color range per percentage**			0	25	50	100

Table S2. SARS-CoV-2 genome single nucleotide variants (SNVs) in postmortem tissues and plasma of case 07. Variants were mapped to clade 20D genome and called if case frequencies were higher than 20% in at least one anatomical compartment. SNV frequency is noted per organ. A heat map with different colors indicates the frequency of mutant detection per organ (the lower bar shows the color range of different percentages). Bold numbers indicate that the latter variant was also identified in SARS-CoV-2 progeny on Vero E6 cells of respective tissue samples (frequencies >1%).

Supplementary Table 3

Gene	Nt change	AA change	Frequency of detection per anatomical compartment (%)					
			Lungs	Plasma	Heart	Kidney	Liver	Spleen
ORF1a	T322A	/	40,81	0	6,57	0	0	0
ORF1a	G1068A	NSP2:G268E	0	0	0	0	0	28,5
ORF1a	C1862T	NSP2:L533F	33,28	5,9	8,78	0	0	3,11
ORF1a	C2094T	NSP2:S610L	**99,56**	**99,55**	**99,62**	**100**	**100**	**99,62**
ORF1a	T2149C	/	20,58	1,35	<1	<1	<1	<1
ORF1a	T5395A	NSP3:F2328V	39,67	5,95	4,12	<1	11,9	4,03
ORF1a	A5405G	NSP3:I1714V	17,73	10,45	30,22	5,76	10,89	4,1
ORF1a	T5406C	NSP3:I1714T	**10,63**	**12,5**	**28,95**	**89,21**	**32,82**	**6,63**
ORF1a	C7239T	NSP3:A2325V	46,19	8,06	20,07	<1	16,67	5,56
ORF1a	T7247G	NSP3:F2328V	**29,24**	**14,66**	**6,78**	**87,36**	**37,5**	**6,77**
ORF1a	C7279T	/	**1,17**	1,84	<1	**83,3**	<1	<1

ORF1a	C8175T	NSP3:A2637V	**14,31**	**45,94**	**42,28**	1,85	**38,91**	**11,11**
ORF1a	A8387G	NSP3:N2708D	**1,92**	**2,89**	<1	**88,86**	2,5	1,32
ORF1a	C9438T	NSP4:T3058I	**14,4**	**44,24**	**24,27**	1,11	**35,56**	**11,64**
ORF1a	C9491T	NSP4:H3076Y	2,84	1,28	33,29	<1	5,65	4,05
ORF1a	A9737G	NSP4:S3158G	**39,58**	**9,44**	**7,07**	**44,79**	**13,87**	5,96
ORF1a	C10369T	/	1,54	<1	<1	<1	6,04	27,3
ORF1a	C11008T	/	20,69	2,92	5,3	<1	3,69	2,17
ORF1a	C12439T	NSP8:P4058L	**8,71**	**5,35**	**5,76**	**48,05**	**21,86**	**4,07**
ORF1a	C12513T	NSP8:T4083M	<1	<1	<1	7,04	<1	**62,73**
ORF1a	T13417C	/	**99,25**	**98,09**	**97,72**	**99,15**	**94,8**	**98,89**
ORF1a	A13433G	NSP10:M4390G	**1,13**	**1,59**	<1	**30,19**	1,84	<1
ORF1b	A13947T	/	**99,5**	**99,17**	**99,46**	**99,26**	**99,64**	**98,96**
ORF1b	C14786T	RdRp:A440V	46,74	4,66	8,13	<1	8,85	3,66
ORF1b	C14937T	/	<1	<1	<1	<1	<1	**64,31**
ORF1b	C15222T	/	1,84	24,59	7,67	1,24	7,97	2,96
ORF1b	C16092T	/	<1	<1	<1	30,42	<1	<1
ORF1b	C17004T	/	39	6,46	13,84	0	7,85	4,12
ORF1b	T18024C	/	<1	<1	<1	27,86	<1	<1
ORF1b	A18179G	NSP14:K1571R	**<1**	**27,26**	**4,86**	<1	**6,34**	**3,35**
ORF1b	T18678C	/	**<1**	**30,76**	**6,55**	<1	**7,03**	**3,55**
ORF1b	T18750C	/	<1	<1	<1	27,13	<1	<1
ORF1b	C18979T	NSP14:H1838Y	<1	<1	<1	40,81	<1	<1
S	C21789T	S:T76I	41,05	7,19	<1	<1	11,52	4,13
S	G22363T	/	**4,65**	2,14	**<1**	**43,97**	3,4	1,06
S	G22661T	S:V367F	**5,62**	**<1**	**10,34**	**3,07**	**1,9**	**68,08**
S	A22920T	S:Y453F	52,52	<1	<1	<1	11,13	5,06
S	A23063T	S:N501Y	**18,46**	**14,29**	**20,69**	**39,63**	**31,27**	**5,04**
S	G23782A	S:M740I	20,87	8,26	<1	<1	4,55	1,72
S	G24316T	S:E918D	**<1**	**3**	**45,65**	<1	**11,33**	**1,94**
S	T24450C	S:V963A	36,09	6,21	16,15	<1	11,27	3,15
S	C24642T	S:T1027I	**44,18**	**27,94**	**27,48**	**49,78**	**23,26**	7,74
E	C26333T	E:T30I	**83,41**	**49,57**	**81,39**	**2,25**	**60,29**	**41,17**
E	C26351T	E:A36V	<1	**<1**	**<1**	<1	<1	**33,61**
ORF7a	A27574T	ORF7a:T61S	**2**	2,1	**<1**	**88,65**	3,16	<1
N	A28336T	/	<1	<1	<1	<1	<1	**62,93**
N	C29200T	/	<1	<1	54,51	<1	<1	<1
N	A29424G	N:Q384R	<1	<1	<1	27,03	<1	<1
ORF10	C29592T	ORF10:T34M	**1,78**	1,45	**1,98**	**49,64**	2,76	<1
3' UTR	G29744A	/	<1	<1	<1	<1	<1	**61,12**
**Color range per percentage**			<1	20	40	60	80	100

Table S3. SARS-CoV-2 genome single nucleotide variants (SNVs) in postmortem tissues and plasma of case 13. The same table as Table 1 is shown as a heat map with different colors indicating the frequency of mutant detection per organ (the lower bar shows the color range of different percentages).

Supplementary Figure 7

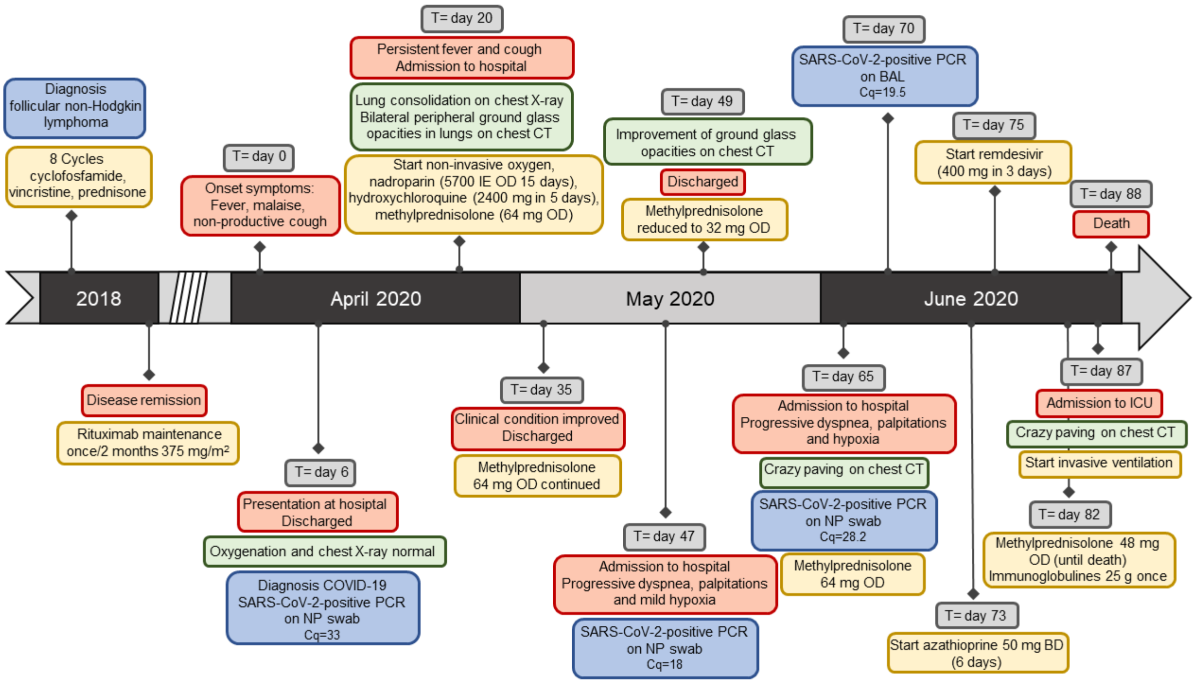

Figure S7. Timeline of the clinical history and disease course in case 13. Abbreviations (in alphabetical order): BAL= broncho-alveolar lavage, BD= twice a day, Cq= quantification cycle, CT= computed tomography, ICU= intensive care unit, NP swab= nasopharyngeal swab, OD= once a day, T= time point.

Supplementary Figure 8

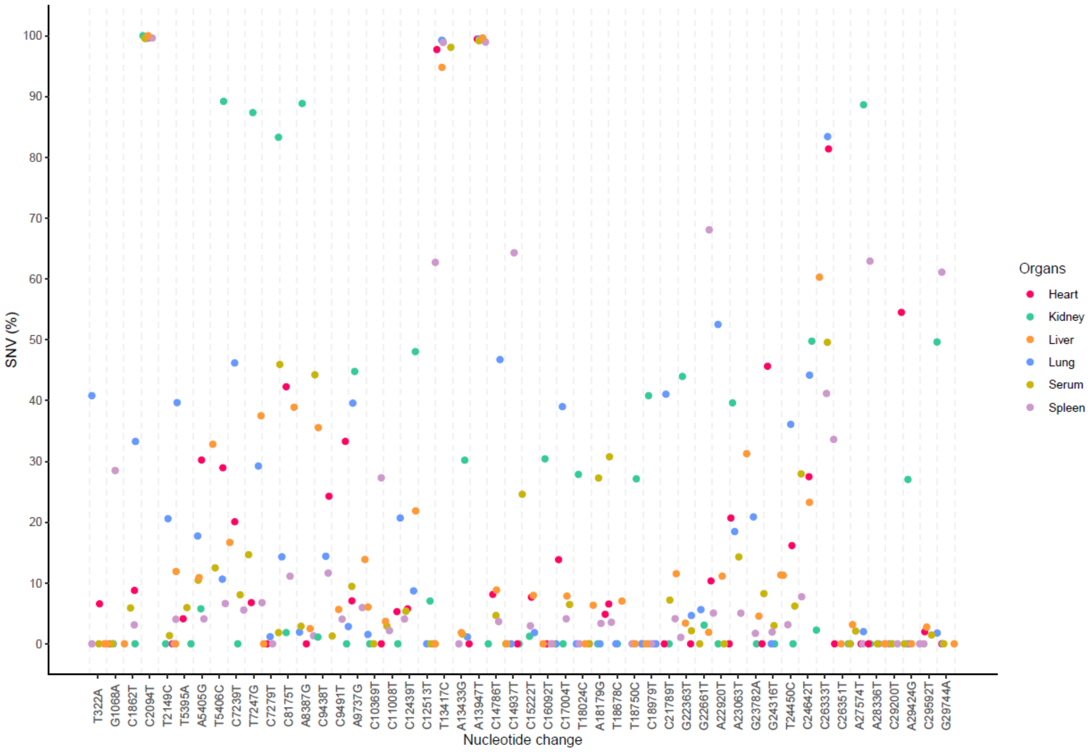

Figure S8. Scatter plot of SARS-CoV-2 SNV frequencies in postmortem tissues and plasma of case 13 ranked per anatomical compartment. Nucleotide positions and single nucleotide variation (SNV) frequencies are indicated on the X axis and the Y axis, respectively. Different colors indicate different organs (shown in the figure legend).

Supplementary Table 4

Gene	Nt position	Size (bp)	Fequency of detection per anatomical compartment					
			Lungs	Plasma	Heart	Kidney	Liver	Spleen
S	21645-23592	1947	<1	<1	<1	<1	<1	19,29
S	21737-23665	1928	<1	<1	<1	12,16	<1	<1
S	21892-23078	1186	<1	<1	<1	<1	52,62	<1
S	22063-22485	422	<1	<1	<1	<1	11,61	<1
ORF8	27998-28089	91	<1	1,27	<1	83,86	10,35	<1
**Color range per percentage**			<1	20	40	60	80	100

Table S4. SARS-CoV-2 genome deletions in postmortem tissues and plasma of case 13. Variants were mapped to clade 20B genome and deletion frequencies were estimated using Sniffles (v1.0.11). A heat map with different colors indicates the frequency of mutant detection per organ (the lower bar shows the color range of different percentages).

Supplementary Figure 9

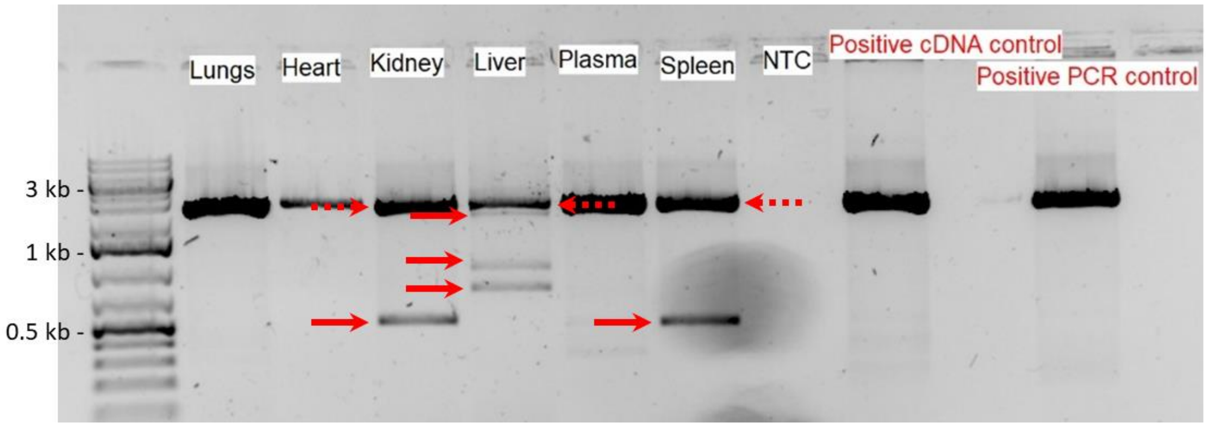

Figure S9. PCR product patterns amplified by the primer pair A6 targeting the partial S gene (nts 21,386-23,824) (case 13). Samples are cDNA derived from different tissues, a negative control (NTC; no template), positive cDNA control (newly synthetized cDNA from a SARS-CoV-2-positive nasopharyngeal swab), or positive PCR control (stored cDNA from a SARS-CoV-2-positive nasopharyngeal swab). Regular arrows indicate products of SARS-CoV-2 variants with large genomic deletions and the dashed arrows indicate the predicted product (2483 bp).

Supplementary Table 5

Name	Forward (5'-3')	Reverse (5'-3')
ACE2	CATTGGAGCAAGTGTTGGATCTT	GAGCTAATGCATGCCATTCTCA
TMPRSS2	ACCGGAAAACCCCTATCCCG	TGCAGACGACGGGGTTGGAA
ACTB	GGCTGTATTCCCCTCCATCG	CCAGTTGGTAACAATGCCATGT
GAPDH	AGGTCGGTGTGAACGGATTTG	GGGGTCGTTGATGGCAACA
YWHA	TGTCACGGTGTGGACGC	ATGACGTCAAACGCTTCTGG
SARS-CoV-2 N1*	GACCCCAAAATCAGCGAAAT	TCTGGTTACTGCCAGTTGAATCTG

Table S5. Primer sequences used for qPCR and ddPCR. *For the SARS-CoV-2 N1 qPCR assay, a TaqMan® probe was used. This probe was labeled at the 5'-end with the reporter molecule 6-carboxyfluorescein (FAM) and with the quencher Black Hole Quencher 1 (BHQ-1) at the 3'-end (Integrated DNA Technologies).

Supplementary Table 6

We gratefully acknowledge the following Authors from the Originating laboratories responsible for obtaining the specimens, as well as the Submitting laboratories where the genome data were generated and shared via GISAID, on which this research is based.

All Submitters of data may be contacted directly via www.gisaid.org

Authors are sorted alphabetically

Accession ID	Originating Laboratory	Submitting Laboratory	Authors
EPI_ISL_1020171, EPI_ISL_1020172, EPI_ISL_1020173, EPI_ISL_1020174	AZ Klina	AZ Klina	Dr. C. Vael
EPI_ISL_1029957, EPI_ISL_1029958, EPI_ISL_1029959, EPI_ISL_1029960, EPI_ISL_1029961, EPI_ISL_1029962, EPI_ISL_1029963, EPI_ISL_1029964, EPI_ISL_1029965, EPI_ISL_1029966, EPI_ISL_1029968, EPI_ISL_1029969, EPI_ISL_1029971, EPI_ISL_1029972, EPI_ISL_1029973			
see above	Cliniques universitaires Saint-Luc	UCLouvain/IREC/MBLG	Jean Ruelle, Lysa Pinsmaye, Benoit Kabamba Mukadi
EPI_ISL_1180935	LHUB-ULB	LHUB-ULB	Charlotte Michel, Olivier Vandenberg, Marie Hallin
EPI_ISL_407976	KU Leuven, Clinical and Epidemiological Virology	KU Leuven, Clinical and Epidemiological Virology	Bert Vanmechelen, Elke Wollants, Annabel Rector, Els Keyaerts, Lies Laenen, Marc Van Ranst, and Piet Maes
EPI_ISL_415153	KU Leuven, Clinical and Epidemiological Virology	KU Leuven, Clinical and Epidemiological Virology	Bert Vanmechelen, Joan Marti-Carreras, Tony Wawina, Marc Van Ranst, Piet Maes
EPI_ISL_415154	KU Leuven, Clincal and Epidemiological Virology	KU Leuven, Clincal and Epidemiological Virology	Bert Vanmechelen, Joan Marti-Careras, Tony Wawina, Marc Van Ranst, Piet Maes.
EPI_ISL_415155	KU Leuven, Clinical and Epidemiological Virology	KU Leuven, Clinical and Epidemiological Virology	Bert Vanmechelen, Joan Marti-Carreras, Tony Wawina, Marc Van Ranst, Piet Maes
EPI_ISL_415156, EPI_ISL_415157, EPI_ISL_415158, EPI_ISL_415159	KU Leuven, Clinical and Epidemiological Virology	KU Leuven, Clinical and Epidemiological Virology	Bert Vanmechelen, Joan Marti-Carreras, Tony Wawina, Piet Maes
EPI_ISL_416467, EPI_ISL_416468, EPI_ISL_416469, EPI_ISL_416470, EPI_ISL_416471, EPI_ISL_416472, EPI_ISL_416475, EPI_ISL_416476	KU Leuven, Clinical and Epidemiological Virology	KU Leuven, Clinical and Epidemiological Virology	Bert Vanmechelen, Tony Wawina, Joan Marti-Carreras, Piet Maes
EPI_ISL_416997, EPI_ISL_417004, EPI_ISL_417006, EPI_ISL_417008, EPI_ISL_417009, EPI_ISL_417012, EPI_ISL_417013, EPI_ISL_417014, EPI_ISL_417015, EPI_ISL_417016, EPI_ISL_417017, EPI_ISL_417018, EPI_ISL_417019, EPI_ISL_417020, EPI_ISL_417021, EPI_ISL_417022, EPI_ISL_417023, EPI_ISL_417025			
see above	Department of Clinical Microbiology	GIGA Medical Genomics	Durkin Keith, Artesi Maria, Bontems Sébastien, Boreux Raphaël, Meex Cécile, Melin Pierrette, Hayette Marie-Pierre, Bours Vincent.
EPI_ISL_417422, EPI_ISL_417424, EPI_ISL_417425, EPI_ISL_417426, EPI_ISL_417427, EPI_ISL_417428, EPI_ISL_417429, EPI_ISL_417430	KU Leuven, Clinical and Epidemiological Virology	KU Leuven, Clinical and Epidemiological Virology	Joan Marti-Carerras, Tony Wawina, Bert Vanmechelen, Piet Maes
EPI_ISL_418270	KU Leuven, Clinical and Epidemiological Virology	KU Leuven, Clinical and Epidemiological Virology	Tony Wawina, Joan Marti-Carreras, Bert Vanmechelen, Piet Maes
EPI_ISL_418624, EPI_ISL_418625, EPI_ISL_418626, EPI_ISL_418627, EPI_ISL_418628, EPI_ISL_418629, EPI_ISL_418630, EPI_ISL_418631, EPI_ISL_418632, EPI_ISL_418633, EPI_ISL_418634, EPI_ISL_418635, EPI_ISL_418636, EPI_ISL_418638, EPI_ISL_418639, EPI_ISL_418640, EPI_ISL_418645, EPI_ISL_418646, EPI_ISL_418648, EPI_ISL_418649, EPI_ISL_418650, EPI_ISL_418651, EPI_ISL_418652, EPI_ISL_418653, EPI_ISL_418654, EPI_ISL_418655, EPI_ISL_418656, EPI_ISL_418657, EPI_ISL_418659, EPI_ISL_418660, EPI_ISL_418661, EPI_ISL_418663, EPI_ISL_418664			
see above	Department of Clinical Microbiology	GIGA Medical Genomics	Keith Durkin, Maria Artesi, Sébastien Bontems, Raphaël Boreux, Cécile Meex, Pierrette Melin, Marie-Pierre Hayette, Vincent Bours.
EPI_ISL_418792, EPI_ISL_418793	KU Leuven, Clinical and Epidemiological Virology	KU Leuven, Clinical and Epidemiological Virology	Bert Vanmechelen, Tony Wawina, Joan Marti-Carreras, Piet Maes
EPI_ISL_418794, EPI_ISL_418795, EPI_ISL_418796, EPI_ISL_418797, EPI_ISL_418798, EPI_ISL_418800, EPI_ISL_418805, EPI_ISL_418806, EPI_ISL_418863, EPI_ISL_418981, EPI_ISL_418982, EPI_ISL_418983, EPI_ISL_418984, EPI_ISL_418985, EPI_ISL_418986, EPI_ISL_418987			
see above	KU Leuven, Clinical and Epidemiological Virology	KU Leuven, Clinical and Epidemiological Virology	Bert Vanmechelen, Joan Marti-Carreras, Tony Wawina, Piet Maes
EPI_ISL_418988	Institute information KU Leuven, Clinical and Epidemiological Virology	Institute information KU Leuven, Clinical and Epidemiological Virology	Bert Vanmechelen, Joan Marti-Carreras, Tony Wawina, Piet Maes
EPI_ISL_418989	KU Leuven, Clinical and Epidemiological Virology	KU Leuven, Clinical and Epidemiological Virology	Bert Vanmechelen, Joan Marti-Carreras, Tony Wawina, Piet Maes
EPI_ISL_419259	Lab voor klinische biologie	Onderzoeksgroep Virologie	Laurens Lambrechts, Nick Vereecke, Marthe Pauwels, Basiel Cole, Bruno Verhasselt, Linos Vandekerckhove, Hans Nauwynck, Sebastiaan Theuns
EPI_ISL_419264	Lab voor klinische biologie	Onderzoeksgroep Virologie	Nick Vereecke, Laurens Lambrechts, Marthe Pauwels, Basiel Cole, Bruno Verhasselt, Linos Vandekerckhove, Hans Nauwynck, Sebastiaan Theuns
EPI_ISL_419265	Lab voor klinische biologie	Onderzoeksgroep Virologie	Laurens Lambrechts, Nick Vereecke, Marthe Pauwels, Basiel Cole, Bruno Verhasselt, Linos Vandekerckhove, Hans Nauwynck, Sebastiaan Theuns
EPI_ISL_419266	Lab voor klinische biologie	Onderzoeksgroep Virologie	Nick Vereecke, Laurens Lambrechts, Marthe Pauwels, Basiel Cole, Bruno Verhasselt, Linos Vandekerckhove, Hans Nauwynck, Sebastiaan Theuns
EPI_ISL_420314, EPI_ISL_420315, EPI_ISL_420316, EPI_ISL_420317, EPI_ISL_420318, EPI_ISL_420319, EPI_ISL_420320, EPI_ISL_420321, EPI_ISL_420322, EPI_ISL_420323, EPI_ISL_420324, EPI_ISL_420325, EPI_ISL_420326, EPI_ISL_420327, EPI_ISL_420328, EPI_ISL_420329, EPI_ISL_420330, EPI_ISL_420331, EPI_ISL_420332, EPI_ISL_420333, EPI_ISL_420334, EPI_ISL_420335, EPI_ISL_420336, EPI_ISL_420337, EPI_ISL_420338, EPI_ISL_420339, EPI_ISL_420340, EPI_ISL_420341, EPI_ISL_420342, EPI_ISL_420343, EPI_ISL_420344, EPI_ISL_420345, EPI_ISL_420346, EPI_ISL_420347, EPI_ISL_420348, EPI_ISL_420349, EPI_ISL_420350, EPI_ISL_420351, EPI_ISL_420352, EPI_ISL_420353, EPI_ISL_420354, EPI_ISL_420355, EPI_ISL_420356, EPI_ISL_420357, EPI_ISL_420358, EPI_ISL_420359, EPI_ISL_420360, EPI_ISL_420361, EPI_ISL_420362, EPI_ISL_420363, EPI_ISL_420364, EPI_ISL_420365, EPI_ISL_420366, EPI_ISL_420367, EPI_ISL_420368, EPI_ISL_420369, EPI_ISL_420370, EPI_ISL_420371, EPI_ISL_420372, EPI_ISL_420373, EPI_ISL_420374, EPI_ISL_420375, EPI_ISL_420376, EPI_ISL_420377, EPI_ISL_420378, EPI_ISL_420379, EPI_ISL_420380, EPI_ISL_420381, EPI_ISL_420382, EPI_ISL_420383, EPI_ISL_420384, EPI_ISL_420385, EPI_ISL_420386, EPI_ISL_420387, EPI_ISL_420388, EPI_ISL_420389, EPI_ISL_420390, EPI_ISL_420391, EPI_ISL_420392, EPI_ISL_420393, EPI_ISL_420394, EPI_ISL_420395, EPI_ISL_420396, EPI_ISL_420397, EPI_ISL_420398, EPI_ISL_420399, EPI_ISL_420400, EPI_ISL_420401, EPI_ISL_420402, EPI_ISL_420403, EPI_ISL_420404, EPI_ISL_420405, EPI_ISL_420406, EPI_ISL_420407, EPI_ISL_420408, EPI_ISL_420409, EPI_ISL_420410, EPI_ISL_420411, EPI_ISL_420412, EPI_ISL_420413, EPI_ISL_420414, EPI_ISL_420415, EPI_ISL_420416, EPI_ISL_420417, EPI_ISL_420418, EPI_ISL_420419, EPI_ISL_420420, EPI_ISL_420421, EPI_ISL_420422, EPI_ISL_420423, EPI_ISL_420424, EPI_ISL_420425, EPI_ISL_420426, EPI_ISL_420427, EPI_ISL_420428, EPI_ISL_420429, EPI_ISL_420430, EPI_ISL_420431, EPI_ISL_420432, EPI_ISL_420433, EPI_ISL_420434, EPI_ISL_420435, EPI_ISL_420436, EPI_ISL_420437, EPI_ISL_420438, EPI_ISL_420439, EPI_ISL_420440, EPI_ISL_420441, EPI_ISL_420442, EPI_ISL_420443, EPI_ISL_420444, EPI_ISL_420445, EPI_ISL_420446, EPI_ISL_420447, EPI_ISL_420448, EPI_ISL_420449, EPI_ISL_420450, EPI_ISL_420451, EPI_ISL_420452, EPI_ISL_420453, EPI_ISL_420454			
see above	KU Leuven, Clinical and Epidemiological Virology	KU Leuven, Clinical and Epidemiological Virology	Joan Marti-Carreras, Bert Vanmechelen, Tony Wawina, Piet Maes
EPI_ISL_421182, EPI_ISL_421183, EPI_ISL_421184, EPI_ISL_421185, EPI_ISL_421186, EPI_ISL_421187, EPI_ISL_421188, EPI_ISL_421189, EPI_ISL_421190, EPI_ISL_421191, EPI_ISL_421192, EPI_ISL_421193, EPI_ISL_421194, EPI_ISL_421195, EPI_ISL_421196, EPI_ISL_421198, EPI_ISL_421200, EPI_ISL_421201, EPI_ISL_421202, EPI_ISL_421203, EPI_ISL_421204, EPI_ISL_421205, EPI_ISL_421206, EPI_ISL_421207, EPI_ISL_421210, EPI_ISL_421212, EPI_ISL_421213, EPI_ISL_421214, EPI_ISL_424629, EPI_ISL_424630, EPI_ISL_424631, EPI_ISL_424633, EPI_ISL_424636, EPI_ISL_424637, EPI_ISL_424640, EPI_ISL_424641, EPI_ISL_424642, EPI_ISL_424643, EPI_ISL_424644, EPI_ISL_424645, EPI_ISL_424646, EPI_ISL_424647, EPI_ISL_424649, EPI_ISL_424650, EPI_ISL_424651, EPI_ISL_424652, EPI_ISL_424653, EPI_ISL_424654, EPI_ISL_424655, EPI_ISL_424657, EPI_ISL_424658, EPI_ISL_424660, EPI_ISL_424661, EPI_ISL_424662, EPI_ISL_424664			
see above	Department of Clinical Microbiology	GIGA Medical Genomics	Keith Durkin, Maria Artesi, Sébastien Bontems, Raphaël Boreux, Cécile Meex, Pierrette Melin, Marie-Pierre Hayette, Vincent Bours.
EPI_ISL_425048, EPI_ISL_425050	Lab voor klinische biologie	Onderzoeksgroep Virologie	Laurens Lambrechts, Nick Vereecke, Marthe Pauwels, Basiel Cole, Bruno Verhasselt, Linos Vandekerckhove, Hans Nauwynck, Sebastiaan Theuns
EPI_ISL_425052, EPI_ISL_425053, EPI_ISL_425054, EPI_ISL_425055	Lab voor klinische biologie	Onderzoeksgroep Virologie	Nick Vereecke, Laurens Lambrechts, Marthe Pauwels, Basiel Cole, Bruno Verhasselt, Linos Vandekerckhove, Hans Nauwynck, Sebastiaan Theuns
EPI_ISL_425056, EPI_ISL_425057, EPI_ISL_425058	Lab voor klinische biologie	Onderzoeksgroep Virologie	Laurens Lambrechts, Nick Vereecke, Marthe Pauwels, Basiel Cole, Bruno Verhasselt, Linos Vandekerckhove, Hans Nauwynck, Sebastiaan Theuns
EPI_ISL_425059, EPI_ISL_425060, EPI_ISL_425061	Lab voor klinische biologie	Onderzoeksgroep Virologie	Nick Vereecke, Laurens Lambrechts, Marthe Pauwels, Basiel Cole, Bruno Verhasselt, Linos Vandekerckhove, Hans Nauwynck, Sebastiaan Theuns
EPI_ISL_425062, EPI_ISL_425063, EPI_ISL_425064	Lab voor klinische biologie	Onderzoeksgroep Virologie	Laurens Lambrechts, Nick Vereecke, Marthe Pauwels, Jozefien De Clercq, Bruno Verhasselt, Linos Vandekerckhove, Hans Nauwynck, Sebastiaan Theuns

EPI_ISL_427349, EPI_ISL_427350, EPI_ISL_427351, EPI_ISL_427352, EPI_ISL_427354, EPI_ISL_427357, EPI_ISL_427358, EPI_ISL_427359, EPI_ISL_427364, EPI_ISL_427366, EPI_ISL_427370, EPI_ISL_427371, EPI_ISL_427372, EPI_ISL_427379, EPI_ISL_427385, EPI_ISL_427388, EPI_ISL_427390			
see above	Department of Clinical Microbiology	GIGA Medical Genomics	Keith Durkin, Maria Artesi, Sébastien Bontems, Raphaël Boreux, Cécile Meex, Pierrette Melin, Marie-Pierre Hayette, Vincent Bours.
EPI_ISL_434347, EPI_ISL_434348, EPI_ISL_434349, EPI_ISL_434351, EPI_ISL_434352, EPI_ISL_434354, EPI_ISL_434355	Lab voor klinische biologie	Onderzoeksgroep Virologie	Laurens Lambrechts, Nick Vereecke, Marthe Pauwels, Jozefien De Clercq, Bruno Verhasselt, Linos Vandekerckhove, Hans Nauwynck, Sebastiaan Theuns
EPI_ISL_434357, EPI_ISL_434360, EPI_ISL_434361, EPI_ISL_434362	Lab voor klinische biologie	Onderzoeksgroep Virologie	Nick Vereecke, Laurens Lambrechts, Marthe Pauwels, Jozefien De Clercq, Bruno Verhasselt, Linos Vandekerckhove, Hans Nauwynck, Sebastiaan Theuns
EPI_ISL_434366, EPI_ISL_434368, EPI_ISL_434369, EPI_ISL_434370, EPI_ISL_434371	Hospital AZ Rivierenland	Institute of Tropical Medicine	Philippe Selhorst, Colin Anthony
EPI_ISL_434372, EPI_ISL_434373, EPI_ISL_434374, EPI_ISL_434375, EPI_ISL_434376, EPI_ISL_434377, EPI_ISL_434378, EPI_ISL_434379, EPI_ISL_434380, EPI_ISL_434381, EPI_ISL_434382, EPI_ISL_434383			
see above	Hospital AZ Rivierenland	Institute of Tropical Medicine	Philippe Selhorst, Colin Anthony,
EPI_ISL_434384	Hospital AZ Rivierenland	Institute of Tropical Medicine	Philippe Selhorst, Colin Anthony
EPI_ISL_447120, EPI_ISL_447121, EPI_ISL_447122, EPI_ISL_447123, EPI_ISL_447124, EPI_ISL_447127, EPI_ISL_447128, EPI_ISL_447129, EPI_ISL_447130, EPI_ISL_447133, EPI_ISL_447134, EPI_ISL_447135, EPI_ISL_447136, EPI_ISL_447137, EPI_ISL_447138, EPI_ISL_447139, EPI_ISL_447140, EPI_ISL_447141, EPI_ISL_447148, EPI_ISL_447152, EPI_ISL_447153, EPI_ISL_447155			
see above	Department of Clinical Microbiology	GIGA Medical Genomics	Keith Durkin, Maria Artesi, Sébastien Bontems, Raphaël Boreux, Cécile Meex, Pierrette Melin, Marie-Pierre Hayette, Vincent Bours.
EPI_ISL_450724, EPI_ISL_450725, EPI_ISL_450726, EPI_ISL_450727, EPI_ISL_450728, EPI_ISL_450729, EPI_ISL_450730, EPI_ISL_450731, EPI_ISL_450732, EPI_ISL_450734, EPI_ISL_450736, EPI_ISL_450737			
see above	Hospital AZ Rivierenland	Institute of Tropical Medicine	Philippe Selhorst, Colin Anthony
EPI_ISL_451164, EPI_ISL_451166, EPI_ISL_451167, EPI_ISL_451168, EPI_ISL_451169, EPI_ISL_451170, EPI_ISL_451173	Lab voor klinische biologie	Onderzoeksgroep Virologie	Laurens Lambrechts, Nick Vereecke, Marthe Pauwels, Jozefien De Clercq, Bruno Verhasselt, Linos Vandekerckhove, Hans Nauwynck, Sebastiaan Theuns
EPI_ISL_451175, EPI_ISL_451177, EPI_ISL_451178, EPI_ISL_451179, EPI_ISL_451180, EPI_ISL_451181	Lab voor klinische biologie	Onderzoeksgroep Virologie	Nick Vereecke, Laurens Lambrechts, Marthe Pauwels, Jozefien De Clercq, Bruno Verhasselt, Linos Vandekerckhove, Hans Nauwynck, Sebastiaan Theuns
EPI_ISL_451935	CUB Hopital Erasme Laboratoire d'Anatomie Pathologique	CUB Hopital Erasme Laboratoire d'Anatomie Pathologique	Prof. Isabelle Salmon, Dr. Nicky D'Haene
EPI_ISL_452140	CUB Hopital Erasme Laboratoire d'Anatomie Pathologique	CUB Hopital Erasme Laboratoire d'Anatomie Pathologique	Isabelle Salmon, Nicky D'Haene
EPI_ISL_452142	CUB Hopital Erasme Laboratoire d'Anatomie Pathologique	CUB Hopital Erasme Laboratoire d'Anatomie Pathologique	Isabelle Salmon, Nikcy D'Haene
EPI_ISL_452148, EPI_ISL_452149	CUB Hopital Erasme Laboratoire d'Anatomie Pathologique	CUB Hopital Erasme Laboratoire d'Anatomie Pathologique	Isabelle Salmon, Nicky D'Haene
EPI_ISL_452150	CUB Hopital Erasme Laboratoire d'Anatomie Pathologique	CUB Hopital Erasme Laboratoire d'Anatomie Pathologique	Prof. Isabelle Salmon, Dr Nicky D'Haene
EPI_ISL_452151	CUB Hopital Erasme Laboratoire d'Anatomie Pathologique	CUB Hopital Erasme Laboratoire d'Anatomie Pathologique	Prof. Isabelle Salmon, Dr.Nikcy D'Haene
EPI_ISL_452152	CUB Hopital Erasme Laboratoire d'Anatomie Pathologique	CUB Hopital Erasme Laboratoire d'Anatomie Pathologique	Prof. Isabelle Salmon, Dr Nicky D'Haene
EPI_ISL_455958, EPI_ISL_455961, EPI_ISL_455962, EPI_ISL_455963, EPI_ISL_455965, EPI_ISL_455966, EPI_ISL_455967, EPI_ISL_455968, EPI_ISL_455969, EPI_ISL_455970, EPI_ISL_455972, EPI_ISL_455973, EPI_ISL_455974, EPI_ISL_455975, EPI_ISL_455976, EPI_ISL_455977, EPI_ISL_455978, EPI_ISL_455979			
see above	Department of Clinical Microbiology	GIGA Medical Genomics	Keith Durkin, Maria Artesi, Sébastien Bontems, Raphaël Boreux, Cécile Meex, Pierrette Melin, Marie-Pierre Hayette, Vincent Bours.
EPI_ISL_458156, EPI_ISL_458157, EPI_ISL_458158, EPI_ISL_458159, EPI_ISL_458160, EPI_ISL_458161, EPI_ISL_458162, EPI_ISL_458163, EPI_ISL_458164, EPI_ISL_458165, EPI_ISL_458166, EPI_ISL_458167, EPI_ISL_458168, EPI_ISL_458169, EPI_ISL_458170, EPI_ISL_458171, EPI_ISL_458172, EPI_ISL_458173, EPI_ISL_458174, EPI_ISL_458175, EPI_ISL_458176, EPI_ISL_458177, EPI_ISL_458178, EPI_ISL_458179, EPI_ISL_458180, EPI_ISL_458181, EPI_ISL_458182, EPI_ISL_458183, EPI_ISL_458184, EPI_ISL_458185, EPI_ISL_458186, EPI_ISL_458187, EPI_ISL_458188, EPI_ISL_458189, EPI_ISL_458190, EPI_ISL_458191, EPI_ISL_458192, EPI_ISL_458193, EPI_ISL_458194, EPI_ISL_458195, EPI_ISL_458196, EPI_ISL_458197, EPI_ISL_458198, EPI_ISL_458199, EPI_ISL_458200, EPI_ISL_458201, EPI_ISL_458202, EPI_ISL_458203, EPI_ISL_458204, EPI_ISL_458205, EPI_ISL_458206, EPI_ISL_458207, EPI_ISL_458208, EPI_ISL_458209, EPI_ISL_458210, EPI_ISL_458211, EPI_ISL_458212, EPI_ISL_458213, EPI_ISL_458214, EPI_ISL_458215, EPI_ISL_458216, EPI_ISL_458217, EPI_ISL_458218, EPI_ISL_458219, EPI_ISL_458220, EPI_ISL_458221, EPI_ISL_458222, EPI_ISL_458223, EPI_ISL_458224, EPI_ISL_458225, EPI_ISL_458226, EPI_ISL_458227, EPI_ISL_458228, EPI_ISL_458229, EPI_ISL_458230, EPI_ISL_458231, EPI_ISL_458232, EPI_ISL_458233, EPI_ISL_458234, EPI_ISL_458235, EPI_ISL_462151, EPI_ISL_462152, EPI_ISL_462153, EPI_ISL_462154, EPI_ISL_462155, EPI_ISL_462156, EPI_ISL_462157, EPI_ISL_462158, EPI_ISL_462159, EPI_ISL_462160, EPI_ISL_462161, EPI_ISL_462162, EPI_ISL_462163, EPI_ISL_462164, EPI_ISL_462165, EPI_ISL_462166, EPI_ISL_462167, EPI_ISL_462168, EPI_ISL_462169, EPI_ISL_462170, EPI_ISL_462171, EPI_ISL_462172, EPI_ISL_462173, EPI_ISL_462174, EPI_ISL_462175, EPI_ISL_462176, EPI_ISL_462177, EPI_ISL_462178, EPI_ISL_462179, EPI_ISL_462180, EPI_ISL_462181, EPI_ISL_462182, EPI_ISL_462183, EPI_ISL_462184, EPI_ISL_462185, EPI_ISL_462186, EPI_ISL_462187, EPI_ISL_462188, EPI_ISL_462189, EPI_ISL_462190, EPI_ISL_462191, EPI_ISL_462192, EPI_ISL_462193, EPI_ISL_462194, EPI_ISL_462195, EPI_ISL_462196, EPI_ISL_462197, EPI_ISL_462198, EPI_ISL_462199, EPI_ISL_462200, EPI_ISL_462201, EPI_ISL_462202, EPI_ISL_462203, EPI_ISL_462204, EPI_ISL_462205, EPI_ISL_462206, EPI_ISL_462207, EPI_ISL_462208, EPI_ISL_462209, EPI_ISL_462210, EPI_ISL_462211, EPI_ISL_462212, EPI_ISL_462213, EPI_ISL_462214, EPI_ISL_462215, EPI_ISL_462216, EPI_ISL_462217, EPI_ISL_462218, EPI_ISL_462219, EPI_ISL_462220, EPI_ISL_462221, EPI_ISL_462222, EPI_ISL_462223, EPI_ISL_462224, EPI_ISL_462225, EPI_ISL_462226, EPI_ISL_462227, EPI_ISL_462228, EPI_ISL_462229, EPI_ISL_462230, EPI_ISL_462231, EPI_ISL_462232, EPI_ISL_462233, EPI_ISL_462234, EPI_ISL_462235, EPI_ISL_462236, EPI_ISL_462237, EPI_ISL_462238, EPI_ISL_462239, EPI_ISL_462240, EPI_ISL_462241, EPI_ISL_462242, EPI_ISL_462243, EPI_ISL_462244, EPI_ISL_462245, EPI_ISL_462246, EPI_ISL_462247, EPI_ISL_462248, EPI_ISL_462249, EPI_ISL_462250, EPI_ISL_462251, EPI_ISL_462252, EPI_ISL_462253, EPI_ISL_462254, EPI_ISL_462255, EPI_ISL_462256, EPI_ISL_462257, EPI_ISL_462258, EPI_ISL_462259, EPI_ISL_462260, EPI_ISL_462261, EPI_ISL_462262, EPI_ISL_462263, EPI_ISL_462264, EPI_ISL_462265, EPI_ISL_462266, EPI_ISL_462267, EPI_ISL_462268, EPI_ISL_462269, EPI_ISL_462270, EPI_ISL_462271, EPI_ISL_462272, EPI_ISL_462273, EPI_ISL_462274, EPI_ISL_462275, EPI_ISL_464065, EPI_ISL_464066, EPI_ISL_464067, EPI_ISL_464068, EPI_ISL_464069, EPI_ISL_464070, EPI_ISL_464071, EPI_ISL_464072, EPI_ISL_464073, EPI_ISL_464074, EPI_ISL_464075, EPI_ISL_464076, EPI_ISL_464077, EPI_ISL_464078, EPI_ISL_464079, EPI_ISL_464080, EPI_ISL_464081, EPI_ISL_464082, EPI_ISL_464083, EPI_ISL_464084, EPI_ISL_464085, EPI_ISL_464086, EPI_ISL_464087, EPI_ISL_464088, EPI_ISL_464089, EPI_ISL_464090			
see above	KU Leuven, Rega Institute, Clinical and Epidemiological Virology	KU Leuven, Rega Institute, Clinical and Epidemiological Virology	Tony Wawina-Bokalanga, Bert Vanmechelen, Joan Marti-Carerras, Piet Maes
EPI_ISL_468727, EPI_ISL_468728, EPI_ISL_468729, EPI_ISL_468730, EPI_ISL_468731, EPI_ISL_468732, EPI_ISL_468733, EPI_ISL_468734, EPI_ISL_468735	Lab voor klinische biologie	Onderzoeksgroep Virologie	Laurens Lambrechts, Nick Vereecke, Marthe Pauwels, Bruno Verhasselt, Linos Vandekerckhove, Hans Nauwynck, Sebastiaan Theuns
EPI_ISL_468736, EPI_ISL_468737, EPI_ISL_468738, EPI_ISL_468739, EPI_ISL_468740, EPI_ISL_468741, EPI_ISL_468743, EPI_ISL_468744, EPI_ISL_468745, EPI_ISL_468746	Lab voor klinische biologie	Onderzoeksgroep Virologie	Nick Vereecke, Laurens Lambrechts, Marthe Pauwels, Bruno Verhasselt, Linos Vandekerckhove, Hans Nauwynck, Sebastiaan Theuns
EPI_ISL_471427, EPI_ISL_471429, EPI_ISL_471430, EPI_ISL_471431, EPI_ISL_471435, EPI_ISL_471436	Department of Clinical Microbiology	GIGA Medical Genomics	Keith Durkin, Maria Artesi, Sébastien Bontems, Raphaël Boreux, Cécile Meex, Axelle Chaslain, Céline Fombellida-Lopez, Pierrette Melin, Marie-Pierre Hayette, Vincent Bours.
EPI_ISL_475060, EPI_ISL_475061, EPI_ISL_475062, EPI_ISL_475063, EPI_ISL_475064, EPI_ISL_475065, EPI_ISL_475066, EPI_ISL_475067, EPI_ISL_475068, EPI_ISL_475069, EPI_ISL_475070			
see above	Lab voor klinische biologie	Onderzoeksgroep Virologie	Laurens Lambrechts, Nick Vereecke, Marthe Pauwels, Bruno Verhasselt, Linos Vandekerckhove, Hans Nauwynck, Sebastiaan Theuns
EPI_ISL_475071, EPI_ISL_475072, EPI_ISL_475073, EPI_ISL_475074, EPI_ISL_475075, EPI_ISL_475076, EPI_ISL_475077, EPI_ISL_475078, EPI_ISL_475079, EPI_ISL_475080, EPI_ISL_475081, EPI_ISL_475082			
see above	Lab voor klinische biologie	Onderzoeksgroep Virologie	Nick Vereecke, Laurens Lambrechts, Marthe Pauwels, Bruno Verhasselt, Linos Vandekerckhove, Hans Nauwynck, Sebastiaan Theuns
EPI_ISL_476941, EPI_ISL_476942, EPI_ISL_476943, EPI_ISL_476944, EPI_ISL_476945, EPI_ISL_476946, EPI_ISL_476947, EPI_ISL_476948, EPI_ISL_476949, EPI_ISL_476950, EPI_ISL_476951, EPI_ISL_476952, EPI_ISL_476953, EPI_ISL_476954, EPI_ISL_476955, EPI_ISL_476956, EPI_ISL_476957, EPI_ISL_476958, EPI_ISL_476959, EPI_ISL_476960, EPI_ISL_476961, EPI_ISL_476962, EPI_ISL_476963, EPI_ISL_476964, EPI_ISL_476965, EPI_ISL_476966, EPI_ISL_476967, EPI_ISL_476968, EPI_ISL_476969, EPI_ISL_476970, EPI_ISL_476971, EPI_ISL_476972, EPI_ISL_476973, EPI_ISL_476974, EPI_ISL_476975, EPI_ISL_476976, EPI_ISL_476977, EPI_ISL_476978, EPI_ISL_476979, EPI_ISL_476980, EPI_ISL_476981, EPI_ISL_476982, EPI_ISL_476983, EPI_ISL_476984, EPI_ISL_476985, EPI_ISL_476986, EPI_ISL_476987, EPI_ISL_476988, EPI_ISL_476989, EPI_ISL_476990, EPI_ISL_476991, EPI_ISL_476992, EPI_ISL_476993, EPI_ISL_476994, EPI_ISL_476995, EPI_ISL_476996, EPI_ISL_476997, EPI_ISL_476998, EPI_ISL_476999, EPI_ISL_477000, EPI_ISL_477001, EPI_ISL_477002, EPI_ISL_477003, EPI_ISL_477004, EPI_ISL_477005, EPI_ISL_477006, EPI_ISL_477007			

see above	KU Leuven, Rega Institute, Clinical and Epidemiological Virology	KU Leuven, Rega Institute, Clinical and Epidemiological Virology	Tony Wawina-Bokalanga, Joan Marti-Carerras, Bert Vanmechelen, Piet Maes
EPI_ISL_481221, EPI_ISL_481222, EPI_ISL_481224, EPI_ISL_481225	Lab voor klinische biologie	Onderzoeksgroep Virologie	Laurens Lambrechts, Nick Vereecke, Marthe Pauwels, Bruno Verhasselt, Linos Vandekerckhove, Hans Nauwynck, Sebastiaan Theuns
EPI_ISL_481227, EPI_ISL_481229, EPI_ISL_481230, EPI_ISL_481231, EPI_ISL_481232, EPI_ISL_481233	Lab voor klinische biologie	Onderzoeksgroep Virologie	Nick Vereecke, Laurens Lambrechts, Marthe Pauwels, Bruno Verhasselt, Linos Vandekerckhove, Hans Nauwynck, Sebastiaan Theuns
EPI_ISL_484697, EPI_ISL_484698, EPI_ISL_484699, EPI_ISL_484700, EPI_ISL_484702, EPI_ISL_484703	Department of Clinical Microbiology	GIGA Medical Genomics	Keith Durkin, Maria Artesi, Sébastien Bontems, Raphaël Boreux, Cécile Meex, Axelle Chaslain, Céline Fombellida-Lopez, Pierrette Melin, Marie-Pierre Hayette, Vincent Bours.
EPI_ISL_487432, EPI_ISL_487433, EPI_ISL_487434, EPI_ISL_487435, EPI_ISL_487436	Queen Astrid Military Hospital	Institute of Tropical Medicine	Philippe Selhorst, Colin Anthony
EPI_ISL_498127, EPI_ISL_498128, EPI_ISL_498129, EPI_ISL_498130, EPI_ISL_498131, EPI_ISL_498132, EPI_ISL_498133, EPI_ISL_498134, EPI_ISL_498135, EPI_ISL_498136, EPI_ISL_498137, EPI_ISL_498138, EPI_ISL_498139, EPI_ISL_498141, EPI_ISL_498143, EPI_ISL_498144, EPI_ISL_498146, EPI_ISL_498628, EPI_ISL_498629			
see above	Department of Clinical Microbiology	GIGA Medical Genomics	Keith Durkin, Maria Artesi, Sébastien Bontems, Raphaël Boreux, Cécile Meex, Axelle Chaslain, Céline Fombellida-Lopez, Pierrette Melin, Marie-Pierre Hayette, Vincent Bours.
EPI_ISL_522349, EPI_ISL_522350	KU Leuven, Rega Institute, Clinical and Epidemiological Virology	KU Leuven, Rega Institute, Clinical and Epidemiological Virology	Tony Wawina-Bokalanga, Joan Marti-Carerras, Bert Vanmechelen, Piet Maes
EPI_ISL_577743, EPI_ISL_577744, EPI_ISL_577746	Dutch COVID-19 response team	Erasmus Medical Center	Bas Oude Munnink, Reina Sikkema, David Nieuwenhuijse, Irina Chestakova, Anne van der Linden, Marjan Boter, Emmanuelle Munger, Corine GeurtsvanKessel, Annemiek van der Eijk, Richard Molenkamp, Marion Koopmans, on behalf of the Dutch national COVID-19 response team.
EPI_ISL_582127	Antwerp University Hospital	Institute of Tropical Medicine	Philippe Selhorst, Colin Anthony
EPI_ISL_634878	Lab voor klinische biologie	Onderzoeksgroep Virologie	Laurens Lambrechts, Nick Vereecke, Marthe Pauwels, Bruno Verhasselt, Linos Vandekerckhove, Hans Nauwynck, Sebastiaan Theuns
EPI_ISL_649154	Queen Astrid Military Hospital	Institute of Tropical Medicine	Philippe Selhorst, Colin Anthony
EPI_ISL_734492, EPI_ISL_734493, EPI_ISL_734503, EPI_ISL_734504, EPI_ISL_734505, EPI_ISL_734506, EPI_ISL_734507, EPI_ISL_734508, EPI_ISL_734509, EPI_ISL_734510, EPI_ISL_734511, EPI_ISL_734512, EPI_ISL_734513, EPI_ISL_734514, EPI_ISL_734515, EPI_ISL_734516, EPI_ISL_734517, EPI_ISL_734518, EPI_ISL_734519, EPI_ISL_734520, EPI_ISL_734521, EPI_ISL_734522, EPI_ISL_734523, EPI_ISL_734524, EPI_ISL_734525, EPI_ISL_734526, EPI_ISL_734527, EPI_ISL_734528, EPI_ISL_734529, EPI_ISL_734530, EPI_ISL_734531, EPI_ISL_734532, EPI_ISL_734533, EPI_ISL_734534, EPI_ISL_734535, EPI_ISL_734536, EPI_ISL_734537, EPI_ISL_734538, EPI_ISL_734539, EPI_ISL_734540, EPI_ISL_734541, EPI_ISL_734542, EPI_ISL_734543, EPI_ISL_734544, EPI_ISL_734545, EPI_ISL_734546, EPI_ISL_734547, EPI_ISL_734548, EPI_ISL_734549, EPI_ISL_734550, EPI_ISL_734551, EPI_ISL_734552, EPI_ISL_734553, EPI_ISL_734554, EPI_ISL_734555, EPI_ISL_734556, EPI_ISL_734557, EPI_ISL_734558, EPI_ISL_734559, EPI_ISL_734560, EPI_ISL_734561, EPI_ISL_734562, EPI_ISL_734563, EPI_ISL_734564, EPI_ISL_734565, EPI_ISL_734566, EPI_ISL_734567, EPI_ISL_734568, EPI_ISL_734569, EPI_ISL_734570, EPI_ISL_734571, EPI_ISL_734572, EPI_ISL_734573, EPI_ISL_734574, EPI_ISL_734575, EPI_ISL_734576, EPI_ISL_734577, EPI_ISL_734578, EPI_ISL_734579, EPI_ISL_734580, EPI_ISL_734581, EPI_ISL_734582, EPI_ISL_734583, EPI_ISL_734584, EPI_ISL_734585, EPI_ISL_734586, EPI_ISL_734587, EPI_ISL_734588, EPI_ISL_734589, EPI_ISL_734590, EPI_ISL_734591, EPI_ISL_734592, EPI_ISL_734593, EPI_ISL_734594, EPI_ISL_734595, EPI_ISL_734596, EPI_ISL_734597, EPI_ISL_734598, EPI_ISL_734599, EPI_ISL_734600, EPI_ISL_734601, EPI_ISL_734602, EPI_ISL_734603, EPI_ISL_734604, EPI_ISL_734605, EPI_ISL_734606, EPI_ISL_734607, EPI_ISL_734608, EPI_ISL_734609, EPI_ISL_734610, EPI_ISL_734611, EPI_ISL_734612, EPI_ISL_734613, EPI_ISL_734614, EPI_ISL_734615, EPI_ISL_734616, EPI_ISL_734617, EPI_ISL_734618, EPI_ISL_734619, EPI_ISL_734620, EPI_ISL_734621, EPI_ISL_734622, EPI_ISL_734623, EPI_ISL_734624, EPI_ISL_734625, EPI_ISL_734626, EPI_ISL_734627, EPI_ISL_734628, EPI_ISL_734629, EPI_ISL_734630, EPI_ISL_734631, EPI_ISL_734632, EPI_ISL_734633, EPI_ISL_734634, EPI_ISL_734635, EPI_ISL_734636, EPI_ISL_734637, EPI_ISL_734638, EPI_ISL_734639, EPI_ISL_734640, EPI_ISL_734641, EPI_ISL_734642, EPI_ISL_734643, EPI_ISL_734644, EPI_ISL_734645, EPI_ISL_734646, EPI_ISL_734647, EPI_ISL_734648, EPI_ISL_734649, EPI_ISL_734650, EPI_ISL_734651, EPI_ISL_734652, EPI_ISL_734653, EPI_ISL_734654, EPI_ISL_734655, EPI_ISL_734656, EPI_ISL_734657, EPI_ISL_734658, EPI_ISL_734659, EPI_ISL_734660, EPI_ISL_734661, EPI_ISL_734662, EPI_ISL_734663, EPI_ISL_734664, EPI_ISL_734665, EPI_ISL_734666, EPI_ISL_734667, EPI_ISL_734668, EPI_ISL_734669, EPI_ISL_734670, EPI_ISL_734671, EPI_ISL_734672, EPI_ISL_734673, EPI_ISL_734674, EPI_ISL_734675, EPI_ISL_734676, EPI_ISL_734677, EPI_ISL_734678, EPI_ISL_734679, EPI_ISL_734680, EPI_ISL_734681, EPI_ISL_734682, EPI_ISL_734683, EPI_ISL_734684, EPI_ISL_734685, EPI_ISL_734686, EPI_ISL_734687, EPI_ISL_734688, EPI_ISL_734689, EPI_ISL_734690, EPI_ISL_734691, EPI_ISL_734692, EPI_ISL_734693, EPI_ISL_734694, EPI_ISL_734695, EPI_ISL_734696, EPI_ISL_734697, EPI_ISL_734698, EPI_ISL_734699, EPI_ISL_734700, EPI_ISL_734701, EPI_ISL_734702, EPI_ISL_734703, EPI_ISL_734704, EPI_ISL_734705, EPI_ISL_734706, EPI_ISL_734707, EPI_ISL_734708, EPI_ISL_734709, EPI_ISL_734710, EPI_ISL_734711, EPI_ISL_734712, EPI_ISL_734713, EPI_ISL_734714, EPI_ISL_734715, EPI_ISL_734716, EPI_ISL_734717, EPI_ISL_734718, EPI_ISL_734719, EPI_ISL_734720, EPI_ISL_734721, EPI_ISL_734722, EPI_ISL_734723, EPI_ISL_734724, EPI_ISL_734725, EPI_ISL_734726, EPI_ISL_734727, EPI_ISL_734728, EPI_ISL_734729, EPI_ISL_734730, EPI_ISL_734731, EPI_ISL_734732, EPI_ISL_734733, EPI_ISL_734734, EPI_ISL_734735, EPI_ISL_734736, EPI_ISL_734737, EPI_ISL_734738, EPI_ISL_734739, EPI_ISL_734740, EPI_ISL_734741, EPI_ISL_734742, EPI_ISL_734743, EPI_ISL_734744, EPI_ISL_734745, EPI_ISL_734746, EPI_ISL_734747, EPI_ISL_734748, EPI_ISL_734749, EPI_ISL_734750, EPI_ISL_734751, EPI_ISL_734752, EPI_ISL_734753, EPI_ISL_734754, EPI_ISL_734755, EPI_ISL_734756, EPI_ISL_734757, EPI_ISL_734758, EPI_ISL_734759, EPI_ISL_734760, EPI_ISL_734761, EPI_ISL_734762, EPI_ISL_734763, EPI_ISL_734764, EPI_ISL_734765, EPI_ISL_734766, EPI_ISL_734767, EPI_ISL_734768, EPI_ISL_734769, EPI_ISL_734770, EPI_ISL_734771, EPI_ISL_734772, EPI_ISL_734773, EPI_ISL_734774, EPI_ISL_734775, EPI_ISL_734776, EPI_ISL_734777, EPI_ISL_734778, EPI_ISL_734779, EPI_ISL_734780, EPI_ISL_734781, EPI_ISL_734782, EPI_ISL_734783, EPI_ISL_734784, EPI_ISL_734785, EPI_ISL_734786, EPI_ISL_734787, EPI_ISL_734798, EPI_ISL_734799, EPI_ISL_734800, EPI_ISL_735249, EPI_ISL_738196, EPI_ISL_738197, EPI_ISL_738198, EPI_ISL_738199, EPI_ISL_738200, EPI_ISL_738201, EPI_ISL_738202, EPI_ISL_738203, EPI_ISL_738204, EPI_ISL_738205, EPI_ISL_738206, EPI_ISL_738207, EPI_ISL_738208, EPI_ISL_738209, EPI_ISL_738210, EPI_ISL_738211, EPI_ISL_738212, EPI_ISL_738213, EPI_ISL_738214, EPI_ISL_738215, EPI_ISL_738216, EPI_ISL_738217, EPI_ISL_738218, EPI_ISL_738219, EPI_ISL_738220			
see above	UZ Leuven, National Reference Laboratory for Coronaviruses, Laboratory Medicine, Leuven, Belgium	KU Leuven, Rega Institute, Clinical and Epidemiological Virology	Tony Wawina-Bokalanga, Joan Marti-Carerras, Bert Vanmechelen, Piet Maes
EPI_ISL_949244, EPI_ISL_949245, EPI_ISL_949246, EPI_ISL_949247	Cliniques universitaires Saint-Luc	UCLouvain/IREC/MBLG	Jean Ruelle, Lysa Pinsmaye, Benoit Kabamba Mukadi
EPI_ISL_949248	Cliniques Saint-Pierre Ottignies	UCLouvain/IREC/MBLG	Jean Ruelle, Lysa Pinsmaye, Benoit Kabamba Mukadi
EPI_ISL_949249, EPI_ISL_949250,EPI_ISL_949252	Cliniques universitaires Saint-Luc	UCLouvain/IREC/MBLG	Jean Ruelle, Lysa Pinsmaye, Benoit Kabamba Mukadi

Table S6. GISAID references used to generate the circular maximum-likelihood phylogenetic tree.

.
