# Supplementary material for: Organ-specific genome diversity of replication-competent SARS-CoV-2
Source: Nat Commun. 2021 Nov 16;12:6612. doi: 10.1038/s41467-021-26884-7 (PMC8595628; doi:10.1038/s41467-021-26884-7)
Supplement: Supplementary file 3 — Reporting Summary [file 41467_2021_26884_MOESM3_ESM.pdf]

nature portfolio

Corresponding author(s): Jolien Van Cleemput

Last updated by author(s): 2021/10/18

# Reporting Summary

Nature Portfolio wishes to improve the reproducibility of the work that we publish. This form provides structure for consistency and transparency in reporting. For further information on Nature Portfolio policies, see our Editorial Policies and the Editorial Policy Checklist.

## Statistics

For all statistical analyses, confirm that the following items are present in the figure legend, table legend, main text, or Methods section.

| n/a | Confirmed | |
|---|---|---|
| ☐ | ☒ | The exact sample size (*n*) for each experimental group/condition, given as a discrete number and unit of measurement |
| ☐ | ☒ | A statement on whether measurements were taken from distinct samples or whether the same sample was measured repeatedly |
| ☐ | ☒ | The statistical test(s) used AND whether they are one- or two-sided<br>*Only common tests should be described solely by name; describe more complex techniques in the Methods section.* |
| ☐ | ☒ | A description of all covariates tested |
| ☐ | ☒ | A description of any assumptions or corrections, such as tests of normality and adjustment for multiple comparisons |
| ☐ | ☒ | A full description of the statistical parameters including central tendency (e.g. means) or other basic estimates (e.g. regression coefficient) AND variation (e.g. standard deviation) or associated estimates of uncertainty (e.g. confidence intervals) |
| ☐ | ☒ | For null hypothesis testing, the test statistic (e.g. *F*, *t*, *r*) with confidence intervals, effect sizes, degrees of freedom and *P* value noted<br>*Give P values as exact values whenever suitable.* |
| ☒ | ☐ | For Bayesian analysis, information on the choice of priors and Markov chain Monte Carlo settings |
| ☒ | ☐ | For hierarchical and complex designs, identification of the appropriate level for tests and full reporting of outcomes |
| ☒ | ☐ | Estimates of effect sizes (e.g. Cohen's *d*, Pearson's *r*), indicating how they were calculated |

*Our web collection on statistics for biologists contains articles on many of the points above.*

## Software and code

Policy information about availability of computer code

| | |
|---|---|
| Data collection | QuantaSoft v1.6.6.0320, LightCycler 480 v1.5.0.39, SoftMax Pro v7.1, MinKnow v21.02.1, BaseSpace Sequence Hub v6.8 |
| Data analysis | qbase+ v3.2, QuantaSoft v1.6.6.0320, Hamamutsu NDP.view v2.9.25, Leica Las X v3.7.2.22383, FastQC v0.11.7, Radius v1.4, Guppy v4.2.2 , ARTIC bioinformatics pipeline v1.1.3, Nanopolish v0.13.2, ARTIC artic_vcf_filter tool v1.1.3, Nextstrain toolkit v6, Varscan2 v2.4.3, NGMLR v0.2.7, Sniffles v1.0.11, IQ-Tree v1.6.9, iTol v6, bbmap v37.99, Cutadapt v3.4, samtools v1.6, R v3.1.1 with package ddpcRquant shiny tool (https://ddpcrquant.ugent.be/), SPSS v27.0<br><br>The used code for genome analyses are publicly available on GitHub (https://github.com/laulambr/sarscov2_intrahost; doi:10.5281/zenodo.5569550). |

For manuscripts utilizing custom algorithms or software that are central to the research but not yet described in published literature, software must be made available to editors and reviewers. We strongly encourage code deposition in a community repository (e.g. GitHub). See the Nature Portfolio guidelines for submitting code & software for further information.

## Data

Policy information about availability of data

All manuscripts must include a data availability statement. This statement should provide the following information, where applicable:

- Accession codes, unique identifiers, or web links for publicly available datasets
- A description of any restrictions on data availability
- For clinical datasets or third party data, please ensure that the statement adheres to our policy

SARS-CoV-2 sequence data that support the findings of this study have been deposited in GISAID with accession codes EPI_ISL_1404134, EPI_ISL_1404133,

EPI_ISL_1404136, EPI_ISL_1404135, EPI_ISL_1404141, EPI_ISL_1404140, EPI_ISL_1404132, EPI_ISL_1404131, EPI_ISL_1404142, EPI_ISL_1404138, EPI_ISL_1404137, EPI_ISL_1404139 (https://www.epicov.org/).
The raw sequencing data is publicly available on the SRA under the following BioProject study ID: PRJNA724859 (https://www.ncbi.nlm.nih.gov/bioproject/PRJNA724859/). All data are also available from the corresponding authors.
All other data generated or analyzed during this study are included in this paper and its Supplementary Information files. Data underlying main and supplementary Figures are provided with this paper as a Source Data file.

# Field-specific reporting

Please select the one below that is the best fit for your research. If you are not sure, read the appropriate sections before making your selection.

[x] Life sciences  [ ] Behavioural & social sciences  [ ] Ecological, evolutionary & environmental sciences

For a reference copy of the document with all sections, see nature.com/documents/nr-reporting-summary-flat.pdf

# Life sciences study design

All studies must disclose on these points even when the disclosure is negative.

| | |
|---|---|
| Sample size | The study describes an autopsy series of 13 COVID-19 cases that were performed during the COVID-19 pandemic (between April 15th and June 30th of 2020). No predefined number of patients were selected, as the intent of the study was discovery. In addition, the number of patients going to autopsy was unpredictable. |
| Data exclusions | No data were excluded from the manuscript. |
| Replication | Every patient represents a single entity. ELISA and RT-qPCR data were obtained in duplicate and averages are shown in the figures. Stainings were performed on 1-5 sections per case and representative images are shown. Virus isolations were performed once on tissue homogenates of different samples per case. Electron microscopy was performed in duplicate on viral isolates from plasma and control samples. All PCRs to amplify whole genomes were performed once, except for the PCR using primer pair A6 targeting the partial S gene, which was performed twice. Whole genome sequencing was performed once on tissue samples and once on virus isolates derived from these tissues using both ONT and Illumina sequencing. |
| Randomization | Only in Figure 1, we stratified cases based on duration of disease (< or > than 20 days of disease after onset of symptoms). |
| Blinding | Investigators were blinded to group allocation by altering case numbers during pathological analysis of tissues. Blinding was not relevant for all other analyses, since there was no randominzation. |

# Reporting for specific materials, systems and methods

We require information from authors about some types of materials, experimental systems and methods used in many studies. Here, indicate whether each material, system or method listed is relevant to your study. If you are not sure if a list item applies to your research, read the appropriate section before selecting a response.

## Materials & experimental systems

| n/a | Involved in the study | |
|---|---|---|
| [ ] | [x] | Antibodies |
| [ ] | [x] | Eukaryotic cell lines |
| [x] | [ ] | Palaeontology and archaeology |
| [x] | [ ] | Animals and other organisms |
| [ ] | [x] | Human research participants |
| [ ] | [x] | Clinical data |
| [x] | [ ] | Dual use research of concern |

## Methods

| n/a | Involved in the study | |
|---|---|---|
| [x] | [ ] | ChIP-seq |
| [x] | [ ] | Flow cytometry |
| [x] | [ ] | MRI-based neuroimaging |

## Antibodies

Antibodies used

1. Rabbit polyclonal anti-SARS-CoV-2 nucleocapsid protein (NP) antibody (40143-T62; Sinobiological, Beijing, China) diluted 1:1000
2. Omnimap-anti-Rabbit HRP (#760-4311, Ventana Medical Systems) undiluted
3. Rabbit anti-human ACE2 antibodies (#ab108252; Abcam) diluted 0.5 µg/mL
4. Rabbit anti-human TMPRSS2 (#ab109131; Abcam) diluted 0.5 µg/mL
5. BrightVision Poly-HRP-Anti Rabbit (#DPVR-55HPR; Immunologic) undiluted
6. Mouse anti-SARS-CoV-2 antibody (#MBS569903; MyBioSource) 1:1000 dilution
7. Rabbit anti-pan cytokeratin antibody (#ab9377; Abcam) 1:100 dilution
8. Rabbit anti-CD14 antibody (#ab183322; Abcam) 1:100 dilution
9. Rabbit anti-ACE2 (#PK-AB718-3217, PromoCell) 1:100 dilution
10. Rabbit anti-ICAM-1 (#ab109361, Abcam) 1:100 dilution

| | |
|---|---|
| | 11. Goat anti-mouse FITC (#F2761, ThermoFisher Scientific) 1:200 dilution<br>12. Donkey anti-rabbit Texas Red (#6800, Abcam) 1:100 dilution<br>13. Donkey anti-goat FITC (#A16006, ThermoFisher Scientific) 1:200 dilution |
| Validation | All antibodies were purchased and have been validated by the respective companies.<br><br>1. https://www.sinobiological.com/antibodies/cov-nucleocapsid-40143-t62<br>2. https://www.biocompare.com/9956-Assay-Kit/1885414-OmniMap-antiRb-HRP/<br>3. https://www.abcam.com/ace2-antibody-ab15348.html<br>4. https://www.abcam.com/tmprss2-antibody-epr3862-ab109131.html<br>5. http://www.immunologic.nl/wp-content/uploads/2020/12/IFU-2.2-Detection-DPVR-AP-1.pdf<br>6. https://www.mybiosource.com/monoclonal-antibody/coronavirus-sars-cov-np/569903<br>7. https://www.abcam.com/wide-spectrum-cytokeratin-antibody-ab9377.html<br>8. https://www.abcam.com/cd14-antibody-sp192-ab183322.html<br>9. https://www.bio-connect.nl/corona-related-products/rabbit-anti-human-angiotensin-converting-enzyme-2-sars-receptor-polyclonal-antibody/pk-ab718-3217/sfid/878335<br>10. https://www.abcam.com/icam1-antibody-epr4776-ab109361.html<br>11. https://www.thermofisher.com/antibody/product/Goat-anti-Mouse-IgG-H-L-Cross-Adsorbed-Secondary-Antibody-Polyclonal/F-2761<br>12. https://www.abcam.com/donkey-rabbit-igg-hl-texas-red--ab6800.html<br>13. https://www.thermofisher.com/antibody/product/Donkey-anti-Goat-IgG-H-L-Cross-Adsorbed-Secondary-Antibody-Polyclonal/A16006 |

# Eukaryotic cell lines

Policy information about cell lines

| | |
|---|---|
| Cell line source(s) | Vero E6 cells (ATCC C1008) |
| Authentication | Vero E6 cells were authenticated by ATCC using STR profiling. |
| Mycoplasma contamination | Cell lines are screened for the presence of mycoplasma by PCR on a weekly basis. VeroE6 cells are free of mycoplasma contamination. |
| Commonly misidentified lines<br>(See ICLAC register) | No commonly misidentified cell lines were used in this study. |

# Human research participants

Policy information about studies involving human research participants

| | |
|---|---|
| Population characteristics | Minimally invasive autopsy was performed on a total of 13 COVID-19 patients at Jessa hospital who succumbed to infection between April 15th and June 30th, 2020. All patients were confirmed for SARS-CoV-2 infection through RT-qPCR analysis performed on nasopharyngeal swabs. The mean age of this cohort was 77 (range 64-85) with 5 females and 8 males. |
| Recruitment | Patients hospitalized at the Jessa Hospital in Hassalt who succumbed to SARS-CoV-2 infection between April 15th and June 30th 2020, and of whom we received consent of the patients' legal representatives, were selected. Self-selection bias or other biases were not relevant or should not have impacted the results of this study. |
| Ethics oversight | Documented approval was obtained from the Ethics Committees of Jessa hospital and Hasselt University (Clinicaltrials.gov identifier: NCT 04366882). |

Note that full information on the approval of the study protocol must also be provided in the manuscript.

# Clinical data

Policy information about clinical studies

All manuscripts should comply with the ICMJEguidelines for publication of clinical research and a completedCONSORT checklist must be included with all submissions.

| | |
|---|---|
| Clinical trial registration | Clinicaltrials.gov identifier NCT 04366882 |
| Study protocol | The study protocol is available from the authors on reasonable request. |
| Data collection | Clinical data in this manuscript were retrospectively explored in terms of the associations with virological analyses of autopsy material. Therefore, no predifined dates or locales were set to collect clinical data. However, upon approval from the patient's relatives, limited clinical data from patients collected at the Jessa Hospital were recovered (e.g., age, sex, medication, duration of disease). |
| Outcomes | In this manuscript we report results of secondary outcomes. These were "to describe the quantity of viral RNA in the different tissues and relate this to the clinical, radiological and histopathological findings" and "to study in detail the disease mechanisms at cellular level (including ACE-2 receptor expression in relation to quantity of viral RNA) in the different tissues". Additionally, several non pre-specified exploratory outcomes were added to the trial and reported here (TMPRSS2 expression, virus isolation, viral genome |

analyses), for which approval was obtained by the Ethical Committee of Jessa Hospital.
